# Hydrogen Peroxide-Oxidative Signaling Enhances Biosynthesis of Specialized Metabolites in *Baccharis conferta* Kunth

**DOI:** 10.3390/ijms27062544

**Published:** 2026-03-10

**Authors:** Norma Elizabeth Moreno-Anzúrez, Celic Sibel Sarmiento-Ramírez, Ana Silvia Gutiérrez-Román, Virginia Medina-Pérez, Luis Rafael Garibay-Castro, Elizabeth Rubio-Rodríguez, Gabriela Trejo-Tapia

**Affiliations:** Instituto Politécnico Nacional, Centro de Desarrollo de Productos Bióticos, Departamento de Biotecnología, Laboratorio de Productos Naturales, Yautepec 62730, Morelos, Mexico; moan.norma@gmail.com (N.E.M.-A.); csarmientor2500@alumno.ipn.mx (C.S.S.-R.); 12gtr.ana@gmail.com (A.S.G.-R.); vmedinap@ipn.mx (V.M.-P.); lgaribayc1400@alumno.ipn.mx (L.R.G.-C.)

**Keywords:** chlorogenic acid, caffeoylquinic acids, *DXS* gene expression, hydrogen peroxide elicitation

## Abstract

Hydrogen peroxide (H_2_O_2_) regulates plant metabolism. This study examined its effect on the biosynthesis of specialized metabolites in *Baccharis conferta*, a medicinal plant rich in phenolics and terpenes. Plants were elicited with 25 µM and 250 µM H_2_O_2_. Phenolic changes were evaluated by total phenolic content (TPC), total flavonoid content (TFC), phenylalanine ammonia-lyase (PAL) activity, and LC-MS analysis of flavonoids and hydroxycinnamic acids. Meanwhile, terpene changes were evaluated by HPTLC, total terpene content (TTC), and expression of the 1-deoxy-D-xylulose-5-phosphate synthase (*Bco-DXS1*) gene. H_2_O_2_ markedly modulated both pathways. Phenolic metabolism was activated, particularly under 25 µM H_2_O_2_, with PAL activity increasing by 52%, TPC by 42%, and TFC by 50% relative to the control. Chemical analysis revealed that five compounds, including chlorogenic acid, differed significantly across treatments. Gene expression analysis showed that 25 µM H_2_O_2_ upregulated *Bco-DXS1* and increased TTC, whereas 250 µM H_2_O_2_ repressed gene expression but still enhanced terpene accumulation. Overall, these results suggest that moderate H_2_O_2_ levels function as a signaling molecule in *B. conferta*, simultaneously boosting phenolic and terpene pathways. This highlights controlled H_2_O_2_ elicitation as an effective biotechnological approach to increase the production of valuable metabolites in medicinal plant cultures.

## 1. Introduction

Plants are constantly exposed to diverse environmental stimuli that can influence their physiology and metabolic profiles. To cope with these conditions, they have evolved complex regulatory systems that integrate stress perception and metabolic reprogramming. A central feature of these responses is the biosynthesis of specialized metabolites, which are crucial for plant defense, adaptation, and signaling. These compounds include a wide range of molecules, such as phenols, terpenes, and alkaloids, many of which possess remarkable pharmacological and industrial potential. Understanding the regulatory mechanisms that control specialized metabolism, especially in non-model medicinal plants, is a key step in harnessing their biosynthetic capacity for biotechnological applications [1].

*Baccharis conferta* Kunth (Asteraceae), a medicinal plant from Mexico, is commonly called “escoba” or “escobillo chino.” It has been used in traditional medicine to treat ailments such as joint pain, convulsions, cramps, toothaches, colds, digestive disorders, and urinary problems. Pharmacological research shows that its extracts exhibit diverse biological activities, including anti-inflammatory, antinociceptive, and anti-arthritic effects. These effects are mainly attributed to its specialized metabolites, especially phenolic compounds like phenolic acids (e.g., di-*O*-caffeoylquinic acids) and flavonoids (e.g., vicenin-2), as well as diterpenes, notably neoclerodane-type terpenes (e.g., kindigiol) [2,3]. Various in vitro micropropagation strategies have been developed for *B. conferta*, ranging from cell morphogenesis to plantlet regeneration [4]. These systems have facilitated the study of the chemical composition of *B. conferta* and its physiological interactions with the hemiparasitic plant *Castilleja tenuiflora* Benth. (Orobanchaceae), highlighting changes in the production of its specialized compounds [5]. However, biotechnological tools to increase the production of its specialized metabolites under in vitro conditions have not yet been explored.

Elicitation is a common technique in plant biotechnology for increasing the production of specialized metabolites in plants of pharmacological interest. Elicitors, whether physical, chemical, or biological, stimulate plant defense signaling pathways, resulting in metabolic changes and enhanced biosynthesis of these compounds. Recognition of elicitors activates stress responses that promote the accumulation of bioactive molecules. These elicitors are generally classified by origin as biotic, abiotic, or hormonal [6,7].

Hydrogen peroxide (H_2_O_2_) is a key reactive oxygen species (ROS) in plants, functioning both as a toxic oxidant and a signaling molecule [8,9]. At low levels, H_2_O_2_ acts as a diffusible messenger that regulates plant growth, development, and defense by affecting redox-sensitive transcription factors and enzyme cascades. In contrast, high concentrations can cause oxidative stress, protein oxidation, and cell damage. The effect of H_2_O_2_ signaling depends on its concentration, exposure duration, and subcellular location [10]. As a signaling molecule, H_2_O_2_ is crucial for elicitor-induced specialized metabolism, transmitting signals from various biotic and abiotic stimuli, including salicylic acid (SA), jasmonic acid (JA), heavy metals, chitosan, and UV light [11,12,13]. It can activate MAPK pathways, calcium-dependent protein kinases, and transcription factors like MYB, WRKY, and bHLH, which regulate defense-related biosynthetic genes [14]. These pathways often lead to the activation of phenylalanine ammonia-lyase (PAL), a key enzyme in the phenylpropanoid pathway. For instance, H_2_O_2_ induction increased total phenolics and antioxidant capacity in *Lens culinaris* [11], *Cicer arietinum* [15], and *Lactuca sativa* [13]. These findings demonstrate H_2_O_2_’s versatile role in regulating redox balance and specialized metabolism.

Terpenes are among the largest groups of specialized metabolites, originating from the plastidial methylerythritol phosphate (MEP) and cytosolic mevalonate (MEV) pathways [16]. The MEP pathway, which produces mono- and diterpenes, begins with 1-deoxy-D-xylulose-5-phosphate synthase (DXS), a key enzyme controlling the flow of carbon toward isoprenoid precursors [17]. *DXS* expression is sensitive to stress and hormonal signals, indicating a potential connection to terpenoid accumulation in many plants. H_2_O_2_ can modulate terpene biosynthesis via redox-dependent regulation of DXS and related enzymes [8,17]. Mild oxidative conditions may boost *DXS* expression, while excessive ROS suppresses plastidial isoprenoid production and shifts metabolism to cytosolic pathways [10,17]. Research on *Castilleja tenuiflora*, *Populus trichocarpa*, and *Nepeta* species shows that H_2_O_2_ exposure alters *DXS* expression and terpene profiles in a dose-dependent manner—low levels promote biosynthesis, whereas high levels inhibit it [18,19,20]. This suggests that H_2_O_2_ acts as a redox modulator, balancing both phenolic and terpenoid pathways. Despite increasing evidence that H_2_O_2_ functions as a signaling molecule in plant defense and metabolism, the mechanisms linking ROS detection to the coordinated activation of phenylpropanoid and terpenoid pathways remain poorly understood, especially in medicinal plants with high biotechnological potential.

Various species within the genus *Baccharis* have been studied mainly for their ethnobotanical uses, phytochemicals, and the antimicrobial and pesticidal properties of their essential oils [21,22,23]. However, *B. conferta* has not been extensively researched in in vitro and elicited systems. Although H_2_O_2_ elicitation has been examined in some plants, its application as an elicitor in *B. conferta* remains limited, and its effects on PAL activity, phenolic compound accumulation, and DXS gene expression in this species remain unknown. No studies have explored how H_2_O_2_ affects these biochemical and genetic responses in *B. conferta*. Considering the biochemical potential of *B. conferta*, understanding these regulatory mechanisms is essential. This research aimed to assess how H_2_O_2_ influences the biosynthesis of phenolic and terpene-like metabolites at two concentrations (25 µM and 250 µM) over different exposure times. The findings present the first evidence linking H_2_O_2_ signaling to specialized metabolism in *B. conferta*. They also improve our understanding of how H_2_O_2_-mediated signaling regulates phenolic and terpenoid pathways. Furthermore, recognizing *Bco-DXS* as a gene allows for metabolic engineering to boost the production of pharmacologically important terpenes.

## 2. Results

### 2.1. Evaluation of Stress Symptoms to Differentiate Elicitation from Oxidative Damage

**Figure 1:**
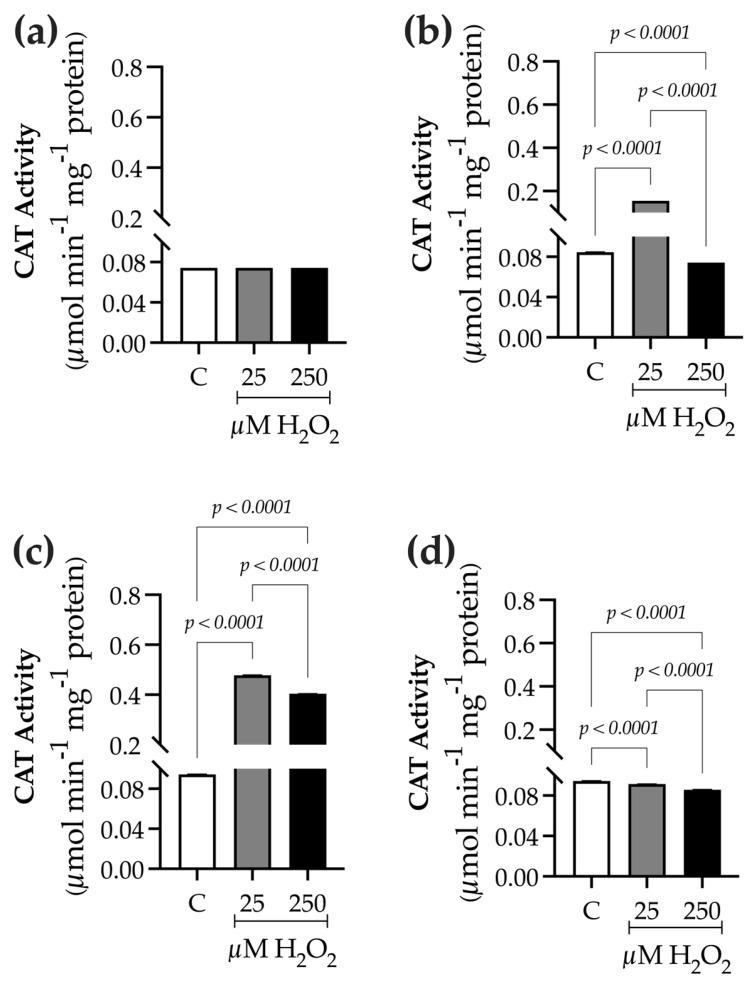
Time-course analysis of catalase (CAT) activity in *Baccharis conferta* plants following hydrogen peroxide (H_2_O_2_) elicitation. (**a**) Baseline (0 h; F = 1.000, *p* = 0.4219, R^2^ = 0.000; K^2^ = 3.600, *p* = 0.1653); (**b**) 9 h after elicitation (F = 54,011, *p* < 0.0001, R^2^ = 1.000; K^2^ = 0.6645, *p* = 0.7173); (**c**) 24 h after elicitation (F = 9293, *p* < 0.0001, R^2^ = 1.000; K^2^ = 1.000, *p* = 0.6064); and (**d**) 48 h after elicitation (F = 3504, *p* < 0.0001, R^2^ = 0.9991; K^2^ = 0.4946, *p* = 0.7809). Data represent mean ± standard error (SE) of three biological replicates (n = 3). Statistical comparisons were performed separately for each time point using one-way ANOVA followed by Tukey’s multiple range test (*p* ≤ 0.05). Data normality was assessed using the D’Agostino–Pearson omnibus test (K^2^). Treatments were defined as C (control), 25, and 250 µM H_2_O_2_.

**Figure 2:**
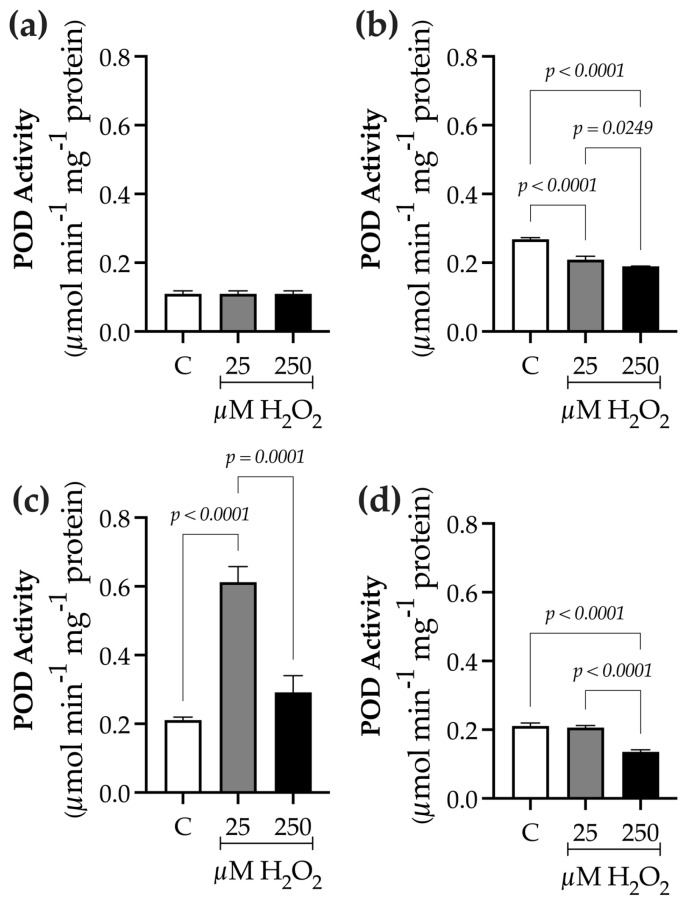
Time-course analysis of peroxidase (POD) activity in *Baccharis conferta* plants following hydrogen peroxide (H_2_O_2_) elicitation. (**a**) Baseline (0 h; F = 1.000, *p* = 0.4219, R^2^ = 0.000; K^2^ = 2.381, *p* = 2.381); (**b**) 9 h after elicitation (F = 119.5, *p* < 0.0001, R^2^ = 0.9755; K^2^ = 1.343, *p* = 0.5110); (**c**) 24 h after elicitation (F = 90.36, *p* < 0.0001, R^2^ = 0.9679; K^2^ = 0.4114, *p* = 0.8114); and (**d**) 48 h after elicitation (F = 115.8, *p* < 0.0001, R^2^ = 0.9747; K^2^ = 1.4939, *p* = 0.4871). Data represent mean ± standard error (SE) of three biological replicates (n = 3). Statistical comparisons were performed separately for each time point using one-way ANOVA followed by Tukey’s multiple range test (*p* ≤ 0.05). Data normality was assessed using the D’Agostino–Pearson omnibus test (K^2^). Treatments were defined as C (control), 25, and 250 µM H_2_O_2_.

To differentiate elicitation effects from possible oxidative damage, responses related to physiological and oxidative stress were assessed after H_2_O_2_ treatment. Visible stress symptoms were recorded at each sampling point (0, 9, 24, and 48 h) and photographed (Supplementary Data S1). No visible damage was observed in plants treated with H_2_O_2_ (25 µM or 250 µM) throughout the 0–48-h period. Compared with the control, treated plants maintained green coloration, tissue turgor, and leaf integrity, with no signs of chlorosis, necrosis, wilting, or tissue collapse. Similarly, root formation and elongation, as well as overall growth, were unaffected under the experimental conditions. Chlorophyll content, as a measure of photosynthetic capacity, was assessed along with fresh biomass (Table 1). Furthermore, the activity levels of key antioxidant enzymes were determined to evaluate the cellular antioxidant defense system, including catalase (Figure 1) and peroxidase (Figure 2).

Throughout the study period, no significant differences in fresh biomass or chlorophyll content were found between H_2_O_2_-treated plants and controls (*p* > 0.05). Initial fresh biomass was 1.69 ± 0.04 g. Overall, mean fresh weight was 1.157 ± 0.234 g for controls, 1.241 ± 0.176 g for 25 µM H_2_O_2_, and 0.983 ± 0.351 g for 250 µM H_2_O_2_. Chlorophyll content averaged 26.62 ± 1.65 µg cm^−2^ at baseline, with control plants at 26.38 ± 1.11 µg cm^−2^, 27.24 ± 2.78 µg cm^−2^ for the 25 µM treatment, and 25.24 ± 1.71 µg cm^−2^ for the 250 µM treatment (Table 1). These findings suggest that hydrogen peroxide treatment did not significantly affect photosynthetic pigment levels in the tested conditions.

Catalase (CAT) activity at baseline (0 h) was 0.07 ± 4.3 × 10^−5^ µmol min^−1^ mg^−1^ protein (Figure 1a). At 9 h after elicitation, control plants exhibited an activity of 0.084 ± 6.1 × 10^−5^ µmol min^−1^ mg^−1^ protein. H_2_O_2_-treated plants showed an increase, reaching 1.8 times higher activity at 25 µM (0.154 ± 1.2 × 10^−4^ µmol min^−1^ mg^−1^ protein) and 0.97 times higher at 250 µM (0.082 ± 9.7 × 10^−5^ µmol min^−1^ mg^−1^ protein) compared to controls (Figure 1b). After 24 h, CAT activity peaked, with a 5.06-fold increase at 25 µM (0.478 ± 5.4 × 10^−4^ µmol min^−1^ mg^−1^ protein) and a 4.3-fold rise at 250 µM (0.403 ± 3.6 × 10^−4^ µmol min^−1^ mg^−1^ protein), while control plants had 0.094 ± 6.0 × 10^−5^ µmol min^−1^ mg^−1^ protein (Figure 1c). At 48 h, control plants maintained an activity of 0.094 ± 6.02 × 10^−5^ µmol min^−1^ mg^−1^ protein. H_2_O_2_ treatments caused a slight increase, reaching 0.96-fold higher activity at 25 µM (0.09 ± 1.1 × 10^−4^ µmol min^−1^ mg^−1^ protein) and 0.90-fold at 250 µM (0.085 ± 1.7 × 10^−4^ µmol min^−1^ mg^−1^ protein) compared to the control (Figure 1d).

POD activity at 0 h (baseline) was 0.10 ± 0.008 µmol min^−1^ mg^−1^ protein (Figure 2a). After 9 h of elicitation, control plants showed an activity of 0.268 ± 0.004 µmol min^−1^ mg^−1^ protein. H_2_O_2_-treated plants displayed increased activity, reaching 0.7 times higher at 25 µM (0.209 ± 0.01 µmol min^−1^ mg^−1^ protein) and 0.90 times higher at 250 µM (0.189 ± 4.6 × 10^−4^ µmol min^−1^ mg^−1^ protein) compared to controls (Figure 2b). At 24 h post-elicitation, the highest POD activity was observed at 25 µM, with a 2.8-fold increase (0.611 ± 0.04 µmol min^−1^ mg^−1^ protein). The 250 µM treatment showed a 0.4-fold increase (0.291 ± 0.04 µmol min^−1^ mg^−1^ protein) relative to the control (0.211 ± 0.008 µmol min^−1^ mg^−1^ protein; Figure 2c). At 48 h, control plants exhibited activity of 0.225 ± 0.0058 µmol min^−1^ mg^−1^ protein. H_2_O_2_ treatments caused slight increases, reaching 0.97 times higher at 25 µM (0.206 ± 0.005 µmol min^−1^ mg^−1^ protein) and 0.65 times higher at 250 µM (0.135 ± 0.006 µmol min^−1^ mg^−1^ protein) compared to controls (Figure 2d).

### 2.2. H_2_O_2_ Modifies TPC, TFC, and PAL Activity

**Figure 3:**
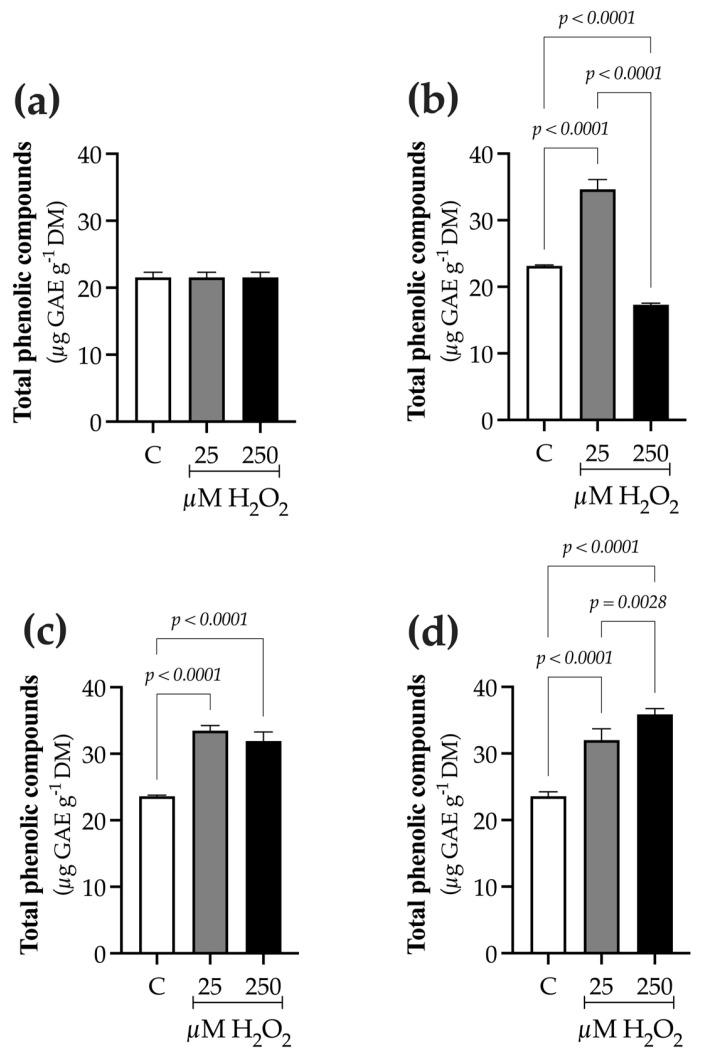
Time-course evaluation of total phenolic compounds (TPC) in *B. conferta* plants following hydrogen peroxide elicitation. TPC was measured at four defined sampling times: (**a**) Baseline (0 h; F = 1.000, *p* = 0.4053, R^2^ = 0.000; K^2^ = 2.805, *p* = 0.2460); (**b**) 9 h after elicitation (F = 422.4, *p* < 0.0001, R^2^ = 0.9895; K^2^ = 4.173, *p* = 0.1241); (**c**) 24 h after elicitation (F = 139.7, *p* < 0.0001, R^2^ = 0.9688; K^2^ = 5.810, *p* = 0.0547); and (**d**) 48 h after elicitation (F = 117.8, *p* < 0.0001, R^2^ = 0.9632; K^2^ = 3.058, *p* = 0.2167). Data represent mean ± standard error (SE) of three biological replicates (n = 3). Statistical comparisons were performed separately for each time point using one-way ANOVA followed by Tukey’s multiple range test (*p* ≤ 0.05). Data normality was assessed using the D’Agostino–Pearson omnibus test (K^2^). GAE: gallic acid equivalents. Treatments were defined as C (control), 25, and 250 µM H_2_O_2_.

Elicitation with both H_2_O_2_ concentrations increased total phenolic compounds (TPC) and total flavonoid content (TFC). TPC levels depended on both exposure time and elicitor concentration (Figure 3). At 0 h, TPC was 21.56 ± 0.7 µg GAE g^−1^ DM (Figure 3a). Plants treated with 25 µM H_2_O_2_ maintained stable TPC levels during the culture period, averaging 34.3 ± 1.3 µg GAE g^−1^ DM. In contrast, at 250 µM H_2_O_2_, TPC significantly dropped at 9 h (17.3 ± 0.2 µg GAE g^−1^ DM; Figure 3b), then gradually recovered, reaching 31.9 ± 1.3 µg GAE g^−1^ DM at 24 h (Figure 3c) and 35.8 ± 0.8 µg GAE g^−1^ DM at 48 h (Figure 3d).

**Figure 4:**
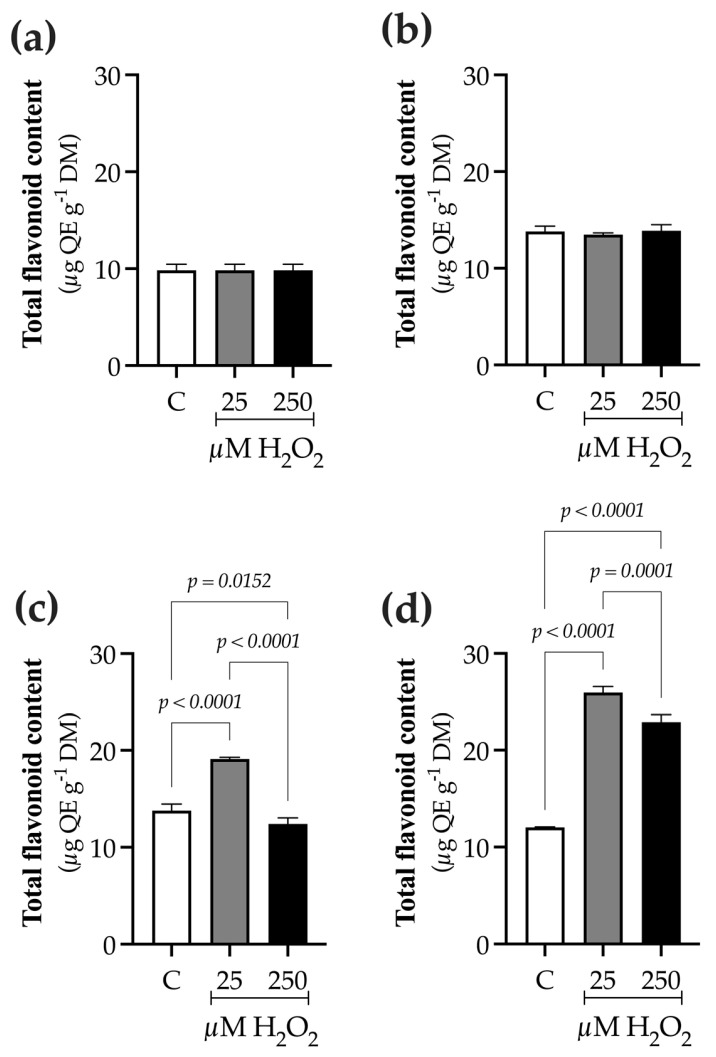
Time-course evaluation of total flavonoid content (TFC) in *B. conferta* plants following hydrogen peroxide (H_2_O_2_) elicitation. TFC was measured at four defined sampling times: (**a**) 0 h (baseline; F = 1.000, *p* = 0.4053, R^2^ = 0.000; K^2^ = 2.062, *p* = 0.3566); (**b**) 9 h after elicitation (F = 0.7025, *p* = 0.5206, R^2^ = 0.1350; K^2^ = 0.5264, *p* = 0.7686); (**c**) 24 h after elicitation (F = 172.3, *p* < 0.0001, R^2^ = 0.9745; K^2^ = 0.3763, *p* = 0.8285); and (**d**) 48 h after elicitation (F = 623.5, *p* < 0.0001, R^2^ = 0.9928; K^2^ = 1.132, *p* = 0.5678). Data represent mean ± standard error (SE) of three biological replicates (n = 3). Statistical comparisons were performed separately for each time point using one-way ANOVA followed by Tukey’s multiple range test (*p* ≤ 0.05). Data normality was assessed using the D’Agostino–Pearson omnibus test (K^2^). QE: quercetin equivalents. Treatments: C = control; 25 and 250 µM H_2_O_2_.

Total flavonoid content (TFC) increased at 24 h post-elicitation in H_2_O_2_-treated plants compared with the control (Figure 4). At 0 h, the TFC was 9.8 ± 0.6 µg RE g^−1^ DM (Figure 4a). At 9 h, a slight increase of approximately 30% was observed across all treatments, reaching 13.7 ± 0.2 µg RE g^−1^ DM (Figure 4b). At 24 h, plants treated with 25 µM H_2_O_2_ showed a 40% increase in TFC (13.7 ± 0.2 µg RE g^−1^ DM), whereas the control and plants treated with 250 µM H_2_O_2_ exhibited similar TFC levels, averaging 13.1 ± 0.04 µg RE g^−1^ DM (Figure 4c). By contrast, at 48 h after elicitation, H_2_O_2_-treated plants exhibited a pronounced increase in TFC at both concentrations, with higher accumulation at 25 µM (25.9 ± 0.7 µg RE g^−1^ DM) than at 250 µM (22.8 ± 0.9 µg RE g^−1^ DM), corresponding to increases of 110% and 90%, respectively, relative to the control (Figure 4d).

**Figure 5:**
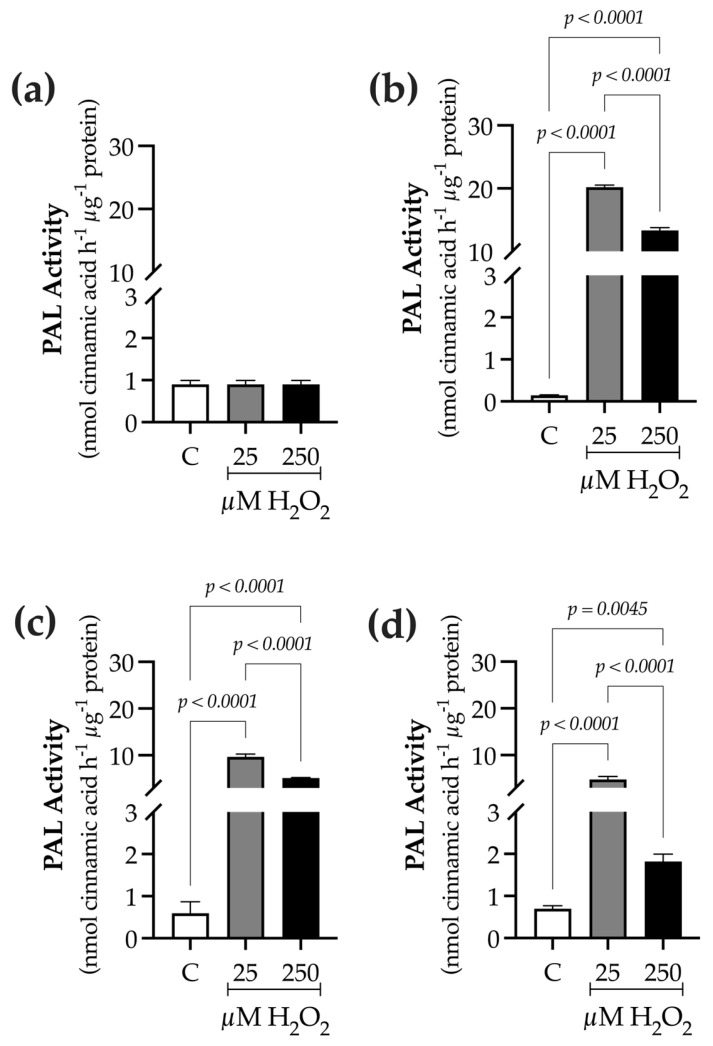
Time-course evaluation of phenylalanine ammonia-lyase (PAL) activity in *B. conferta* plants following elicitation with hydrogen peroxide. Enzyme activity was measured at: (**a**) 0 h (baseline; F = 1.000, *p* = 0.4053, R^2^ = 0.000; K^2^ = 2.651, *p* = 0.2657); (**b**) 9 h after elicitation (F = 4180, *p* < 0.0001, R^2^ = 0.9989; K^2^ = 4.728, *p* = 0.0940); (**c**) 24 h after elicitation (F = 629.1, *p* < 0.0001, R^2^ = 0.9929; K^2^ = 2.782, *p* = 0.2488); and (**d**) 48 h after elicitation (F = 137.8, *p* < 0.0001, R^2^ = 0.9684; K^2^ = 1.132, *p* = 0.5678). Data represent mean ± SE of three independent biological replicates (n = 3). Statistical comparisons were performed separately for each time point using one-way ANOVA followed by Tukey’s multiple range test (*p* ≤ 0.05). Data normality was assessed using the D’Agostino–Pearson omnibus test (K^2^). Treatments were defined as C (control), 25, and 250 µM H_2_O_2_.

PAL enzyme activity was higher at a lower elicitor concentration: 25 µM H_2_O_2_ > 250 µM H_2_O_2_ (Figure 5). At 0 h, PAL activity averaged 0.9 ± 0.09 nmol min^−1^ µg^−1^ protein (Figure 5a). At 9 h, PAL activity reached its maximum in H_2_O_2_-treated plants, with values of 20.2 ± 0.3 nmol min^−1^ µg^−1^ protein at 25 µM H_2_O_2_ and 13.3 ± 0.4 nmol min^−1^ µg^−1^ protein at 250 µM H_2_O_2_, corresponding to 137- and 90-fold increases, respectively, over the control (Figure 5b). As exposure time increased, PAL activity declined. At 24 h, PAL activity remained 15-fold (25 µM H_2_O_2_: 9.6 ± 0.5 nmol min^−1^ µg^−1^ protein) and 8-fold (250 µM H_2_O_2_: 5.1 ± 0.08 nmol min^−1^ µg^−1^ protein) higher than the control (0.5 ± 0.02 nmol min^−1^ µg^−1^ protein) (Figure 5c). At 48 h, PAL activity was still elevated, being 6-fold higher in plants treated with 25 µM H_2_O_2_ (4.8 ± 0.5 nmol min^−1^ µg^−1^ protein) and 1.5-fold higher in plants treated with 250 µM H_2_O_2_ (1.7 ± 0.05 nmol min^−1^ µg^−1^ protein) compared with the control (0.7 ± 0.02 nmol min^−1^ µg^−1^ protein) (Figure 5d).

### 2.3. H_2_O_2_-Induced Changes in the Relative Abundance of Phenolic Acids and Flavonoids and Chlorogenic Acid Concentration

LC–MS analysis of in vitro cultures of *B. conferta* identified nine putative compounds, including four flavonoids—among them vicenin-2, reported here for the first time in in vitro cultures—and five phenolic acids: two caffeoylquinic acids (including chlorogenic acid) and three di-*O*-caffeoylquinic acids (Supplementary Data S2).

**Figure 6:**
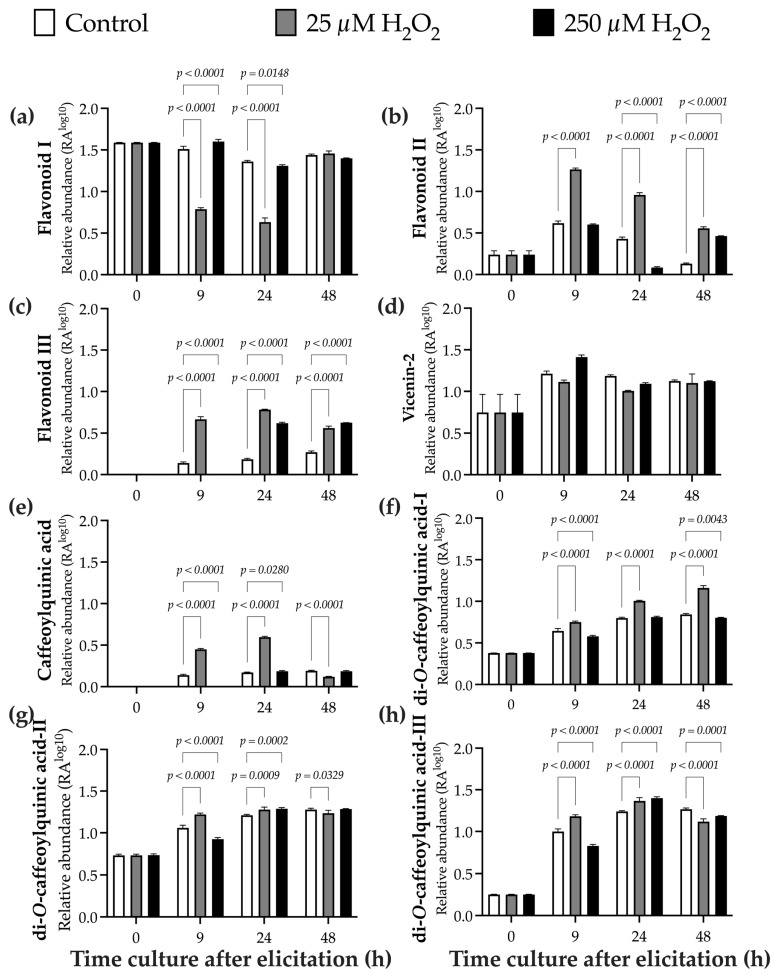
Time-course evaluation of phenolic compounds detected by LC–MS in *B. conferta* plants, after elicitation with H_2_O_2_. Relative abundance (RA^log10^): (**a**) Flavonoid-I (Interaction: F = 389.5, *p* < 0.0001; Time: F = 770.5, *p* < 0.0001; Treatment: F = 1028, *p* < 0.0001; K^2^ = 3.419, *p* = 0.1809); (**b**) Flavonoid-II (Interaction: F = 224.1, *p* < 0.0001; Time: F = 747.6, *p* < 0.0001; Treatment: F = 863.6, *p* < 0.0001; K^2^ = 0.6740, *p* = 0.7139); (**c**) Flavonoid-III (Interaction: F = 690.6, *p* < 0.0001; Time: F = 2824, *p* < 0.0001; Treatment: F = 2010, *p* < 0.0001; K^2^ = 1.643, *p* = 0.3567); and (**d**) vicenin-2 (Interaction: F = 81.24, *p* < 0.0001; Time: F = 260.2, *p* < 0.0001; Treatment: F = 80.59, *p* < 0.0001; K^2^ = 1.138, *p* = 0.5661); (**e**) Caffeoylquinic acid (Interaction: F = 2463, *p* < 0.0001; Time: F = 3347, *p* < 0.0001; Treatment: F = 6060, *p* < 0.0001; K^2^ = 1.475, *p* = 0.4783); (**f**) di-*O*-caffeoylquinic acid-I (Interaction: F = 105.0, *p* < 0.0001; Time: F = 2884, *p* < 0.0001; Treatment: F = 590.0, *p* < 0.0001; K^2^ = 4.322, *p* = 0.1152); (**g**) di-*O*-caffeoylquinic acid-II (Interaction: F = 48.07, *p* < 0.0001; Time: F = 1309, *p* = 0.0001; Treatment: F = 26.77, *p* < 0.0001; K^2^ = 0.1586, *p* = 0.9238); and (**h**) di-*O*-caffeoylquinic acid-III (Interaction: F = 92.23, *p* < 0.0001; Time: F = 4840, *p*= 0.0001; Treatment: F = 28.01, *p* < 0.0001; K^2^ = 3.077, *p* = 0.2147). Measurements were performed at four defined sampling times: 0 h (baseline), 9 h, 24 h, and 48 h after treatment. Data represent mean ± standard error (SE) of three biological replicates (n = 3). Statistical analysis was conducted using two-way ANOVA (treatment × time) followed by Dunnett’s multiple comparison test (*p* ≤ 0.05). A significant interaction between treatment and time was observed for all evaluated metabolites (*p* < 0.0001). Normality was assessed using the D’Agostino–Pearson omnibus test (K^2^). See Supplementary Data S3 for compound identification details.

Figure 6 displays the relative abundance (RA^log10^) of eight detected metabolites, including four flavonoids (Figure 6a–d), one caffeoylquinic acid (Figure 6e), and three di-*O*-caffeoylquinic acids (Figure 6f–h). Although significant differences were observed among treatments for all compounds, flavonoid-I (Figure 6a), flavonoid-II (Figure 6b), flavonoid-III (Figure 6c), caffeoylquinic acid (Figure 6e), and di-*O*-caffeoylquinic acids (Figure 6f–h) were identified as differentially accumulating metabolites (DAMs), as their fold changes were ≥1.5 or ≤0.5.

At 0 h, the RA of flavonoid-I was 1.6 ± 0.01 RA^log10^. Plants treated with 25 µM H_2_O_2_ showed a significant 0.5-fold decrease at 9 h (0.79 ± 0.02 RA^log10^) and 24 h (0.63 ± 0.05 RA^log10^) compared with the control (9 h: 1.51 ± 0.03 RA^log10^ and 24 h: 1.36 ± 0.01 RA^log10^). Plants treated with 250 µM H_2_O_2_ throughout the entire growing period showed values similar to the control, with an average of 1.44 ± 0.14 RA^log10^ (Figure 6a). The relative abundance of flavonoid-II at 0 h was 0.24 ± 0.05 RA^log10^. The control showed 0.62 ± 0.03 RA^log10^ at 24 h, 0.43 ± 0.02 RA^log10^ at 24 h, and 0.13 ± 0.01 RA^log10^ at 48 h. A significant increase was observed in plants treated with H_2_O_2_ (25 µM). At 9 h, flavonoid-II accumulation increased by 2.0-fold (1.27 ± 0.02 RA^log10^), at 48 h by 2.2-fold (0.96 ± 0.03 RA^log10^), and at 24 h by 4.2-fold (0.56 ± 0.02 RA^log10^) (Figure 6b).

At the beginning of the culture, flavonoid-III was not detected at 0 h. In control plants, the relative abundance was 0.14 ± 0.01 RA^log10^ at 9 h, 0.68 ± 0.01 RA^log10^ at 24 h, and 0.27 ± 0.01 RA^log10^ at 48 h. Plants treated with 25 µM H_2_O_2_ showed an increase in relative abundance over time. At 9 h, flavonoid-III accumulation increased by 4.8-fold (0.66 ± 0.03 RA^log10^), followed by slight increases at 24 h (1.1-fold; 0.68 ± 0.01 RA^log10^) and at 48 h (2.1-fold; 0.56 ± 0.02 RA^log10^). Plants treated with 250 µM H_2_O_2_ exhibited a significant increase from 24 h (0.62 ± 0.01 RA^log10^) and 48 h after elicitation (2.3-fold; 0.62 ± 0.001 RA^log10^) compared with the control (Figure 6c). The relative abundance of vicenin-2 at 0 h was 1.29 ± 0.02 RA^log10^. Across all treatments, vicenin-2 RA ^log10^ showed a similar pattern (Figure 6d). The caffeoylquinic acid was not detected at 0 h. Over time, the RA in the control treatment remained constant, averaging 0.16 ± 0.02 RA^log10^. Plants treated with 25 µM H_2_O_2_ showed an increase at 9 h (0.45 ± 0.01 RA^log10^) and 24 h (0.60 ± 0.01 RA^log10^), with 3.2- and 4.3-fold increases, respectively. In contrast, in plants treated with the higher H_2_O_2_ concentration (250 µM), RA of caffeoylquinic acid was 0.19 ± 0.01 RA^log10^ at 24 h and 48 h after elicitation (Figure 6e). The relative abundance of the di-*O*-caffeoylquinic acids remained constant over time. At 0 h, relative abundance was 0.28 ± 0.02 RA^log10^, 0.73 ± 0.01 RA^log10^, and 0.25 ± 0.001 RA^log10^, for di-*O*-caffeoylquinic acid I, II, and III, respectively. In all treatments evaluated, an increase was observed from 9 h after elicitation until the end of the culture. For di-*O*-caffeoylquinic acid I, over time, the increase was 2-fold, with an average of 0.76 ± 0.08 RA^log10^ (Figure 6f). The increase was 1.6-fold (1.2 ± 0.01 RA^log10^) for di-*O*-caffeoylquinic acid II (Figure 6g) and 4.6-fold (1.1 ± 0.1 RA^log10^) for di-*O*-caffeoylquinic acid III (Figure 6h).

**Figure 7:**
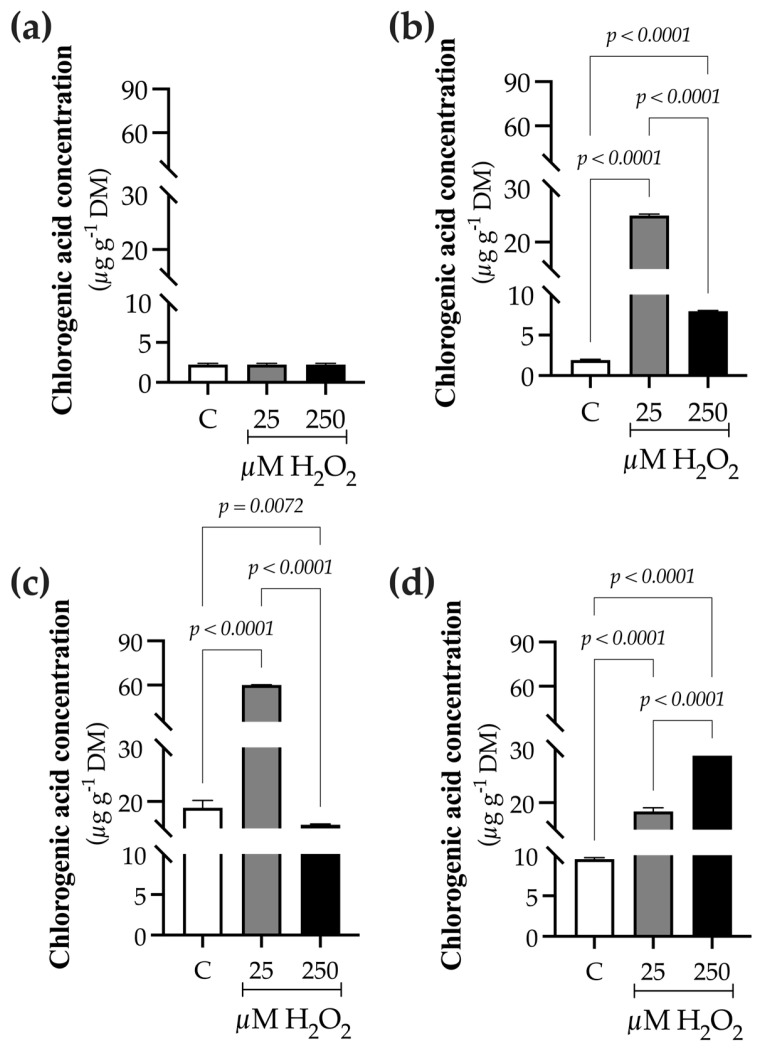
Effect of hydrogen peroxide (H_2_O_2_) on the concentration of chlorogenic acid in *B. conferta* plants. Samples were collected at four defined sampling times: (**a**) 0 h (baseline; F = 1.000, *p* = 0.4219, R^2^ = 0.000; K^2^ = 3.200, *p* = 0.1481); (**b**) 9 h after elicitation (F = 12,970, *p* < 0.0001, R^2^ = 0.9998; K^2^ = 2.703, *p* = 0.2588); (**c**) 24 h after elicitation (F = 2868, *p* < 0.0001, R^2^ = 0.9990; K^2^ = 8.210, *p* = 0.0788); and (**d**) 48 h after elicitation (F = 1838, *p* < 0.0001, R^2^ = 0.9984; K^2^ = 4.173, *p* = 0.1241). Chlorogenic acid content is expressed as µg g^−1^ dry weight. Data represent mean ± standard error (SE) of three biological replicates (n = 3). Statistical comparisons were performed independently for each time point using one-way analysis of variance (ANOVA), followed by Tukey’s multiple comparison test (*p* ≤ 0.05). Normality was assessed using the D’Agostino–Pearson omnibus test (K^2^). See Supplementary Data S4 for calibration curve details. Treatments were defined as C (control), 25, and 250 µM H_2_O_2_.

Elicited treatments also increased chlorogenic acid levels compared to the control (Figure 7). At 0 h, chlorogenic acid concentration was 2.24 ± 0.13 µg g^−1^ DM (Figure 7a). The peak accumulation occurred at 9 h in plants treated with H_2_O_2_, with a 13.0-fold increase at 25 µM H_2_O_2_ (24.9 ± 0.28 µg g^−1^ DM) and a 4.1-fold increase at 250 µM H_2_O_2_ (7.94 ± 0.09 µg g^−1^ DM), both relative to the control (1.91 ± 0.1 µg g^−1^ DM) (Figure 7b). By 24 h, control plants had a chlorogenic acid concentration of 18.85 ± 1.37 µg g^−1^ DM. In contrast, plants treated with H_2_O_2_ showed a 3.6-fold increase at 25 µM (68.40 ± 14.4 µg g^−1^ DM) and a 0.84-fold increase at 250 µM (15.71 ± 0.1 µg g^−1^ DM) compared to the control (Figure 7c). After 48 h, chlorogenic acid levels remained slightly higher in plants exposed to 25 µM H_2_O_2_ (1.3-fold; 18.4 ± 0.6 µg g^−1^ DM), with a more notable increase at 250 µM H_2_O_2_ (2.0-fold; 28.7 ± 0.01 µg g^−1^ DM), relative to the control (13.8 ± 0.4 µg g^−1^ DM) (Figure 7d). Based on fold-change values, chlorogenic acid also matches a DAM.

### 2.4. The Bco-DXS Sequence Is Part of the DXS-1 Family

A partial sequence of the *1-deoxy-D-xylulose-5-phosphate synthase* (*DXS*) gene specific to *B. conferta* was identified and deposited in the NCBI database (https://www.ncbi.nlm.nih.gov/) under accession number OP047919. The sequence consisted of 591 nucleotides and encoded a polypeptide of 197 amino acids (Supplementary Data S5). For bioinformatic analysis, 72 protein sequences from the three known DXS families were used: 34, 25, and 12 sequences corresponding to families I, II, and III, respectively (Supplementary Data S6). The *Bco-DXS* sequence showed strong homology with all analyzed family I sequences. Identity analysis revealed high sequence identity (>80%) with DXS family I proteins from *Gardenia jasminoides* (82%), *Camellia sinensis* (83%), *Magnolia champaca* (84%), and *Taraxacum koksaghyz* (86%), the latter belonging to the Asteraceae family (Supplementary Data S7).

### 2.5. Low H_2_O_2_ Levels Increase Bco-DXS1 Expression

**Figure 8:**
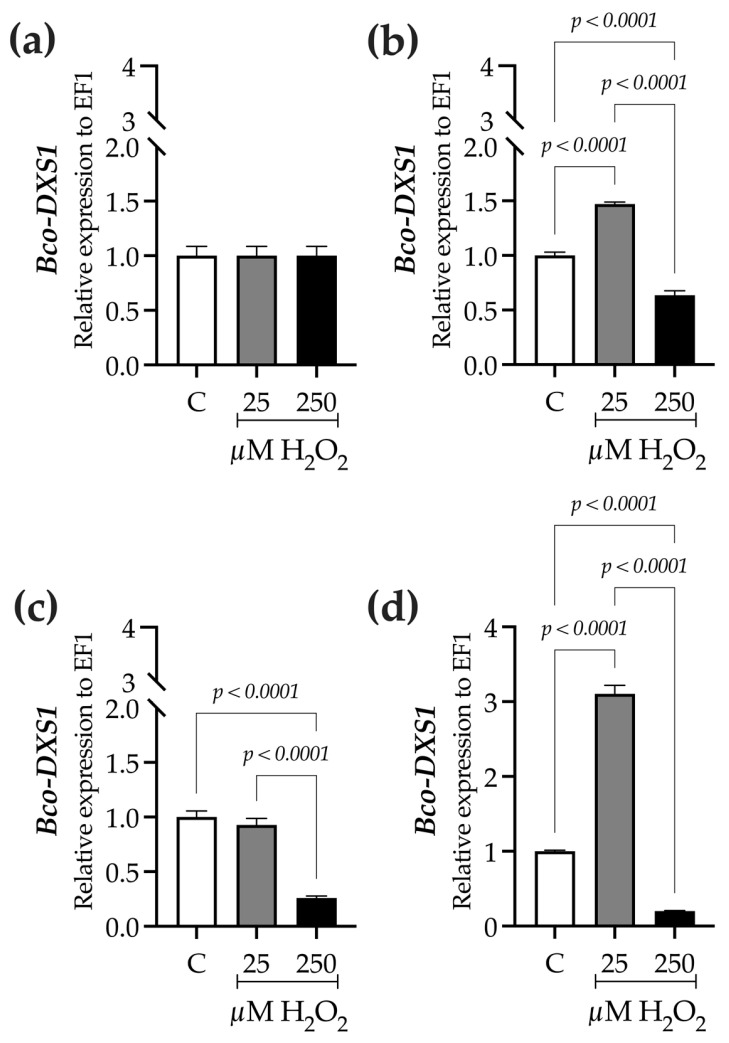
Effect of H_2_O_2_ on the relative expression of 1-deoxy-D-xylulose-5-phosphate synthase (*Bco-DXS1*) gene in *B. conferta* plants. Gene expression was measured at four specific sampling times, each corresponding to different exposure durations. (**a**) 0 h (baseline; F = 1.000, *p* = 0.40219, R^2^ = 0.000; K^2^ = 2.386, *p* = 0.3033); (**b**) 9 h after elicitation (F = 530.9, *p* < 0.0001, R^2^ = 0.9944; K^2^ = 0.6956, *p* = 0.7062); (**c**) 24 h after elicitation (F = 228.0, *p* < 0.0001, R^2^ = 0.9870; K^2^ = 0.1355, *p*= 0.9345); and (**d**) 48 h after elicitation (F = 1553, *p* < 0.0001, R^2^ = 0.9981; K^2^ = 4.540, *p* = 0.1033). Relative expression levels were calculated using the Pfaffl method. Data represent mean ± standard error (SE) of three biological replicates (n = 3). Statistical comparisons were performed separately for each time point using one-way ANOVA followed by Tukey’s multiple range test (*p* ≤ 0.05). Data normality was assessed using the D’Agostino–Pearson omnibus test (K^2^). Treatments were defined as C (control), 25, and 250 µM H_2_O_2_.

Analysis of Bco-DXS1 gene expression and total terpenes content (TTC) across different treatments indicated that *Bco-DXS1* was mainly overexpressed in plants exposed to lower H_2_O_2_ levels. At 9 h, relative expression increased 1.5-fold in plants treated with 25 µM H_2_O_2_, whereas a smaller 0.6-fold induction was observed at 250 µM H_2_O_2_ compared with the control (Figure 8b). By 24 h, *Bco-DXS1* expression remained somewhat elevated, with 0.9- and 0.3-fold increases in plants treated with 25 and 250 µM H_2_O_2_, respectively, relative to the control (Figure 8c). The peak expression occurred at 48 h, when it reached about twice the level of the control in plants treated with 25 µM H_2_O_2_, whereas only a slight 0.2-fold rise was observed at 250 µM H_2_O_2_ (Figure 8d).

**Figure 9:**
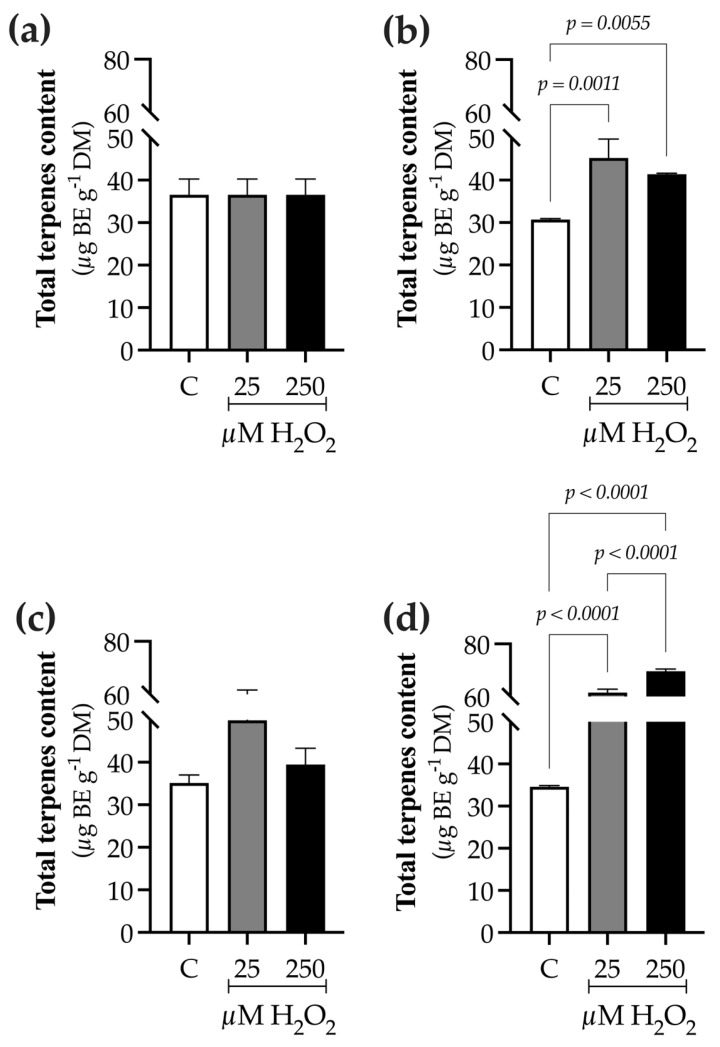
Effect of hydrogen peroxide (H_2_O_2_) on total terpenes content (TTC) in *B. conferta* plants. Samples were collected at four defined sampling times: (**a**) 0 h (baseline; F = 1.000, *p* = 0.4219, R^2^ = 0.000; K^2^ = 3.624, *p* = 0.1633); (**b**) 9 h after elicitation (F = 25.55, *p* = 0.0012, R^2^ = 0.8949; K^2^ = 5.118, *p* = 0.0774); (**c**) 24 h after elicitation (F = 3.218, *p* = 0.1123, R^2^ = 0.5176; K^2^ = 2.469, *p* = 0.2910); and (**d**) 48 h after elicitation (F = 1213, *p* < 0.0001, R^2^ = 0.9975; K^2^ = 1.954, *p* = 0.3765). TTC values are expressed as bacchofertin equivalents (BE). Data represent mean ± standard error (SE) of three biological replicates (n = 3). Statistical comparisons were performed separately for each time point using one-way ANOVA followed by Tukey’s multiple comparison test (*p* ≤ 0.05). Data normality was assessed using the D’Agostino–Pearson omnibus test (K^2^). Treatments were defined as C (control), 25, and 250 µM H_2_O_2_.

The significant increase in *Bco-DXS1* expression in plants exposed to the lowest elicitor concentration (25 µM) was also reflected in the total terpene content (Figure 9). The TTC content at 0 h was 36.5 ± 3.6 µg BE g^−1^ DM (Figure 9a). At 9 h, TTC increased in H_2_O_2_-treated plants, showing a 1.4-fold increase at 25 µM (45.22 ± 1.47 µg BE g^−1^ DM) and a 1.35-fold increase at 250 µM (41.39 ± 0.26 µg BE g^−1^ DM) compared to the control (30.72 ± 0.19 µg BE g^−1^ DM) (Figure 9b). At 24 h, TTC content in control plants increased to 35.11 ± 1.92 µg BE g^−1^ DM. In contrast, TTC levels in H_2_O_2_-treated plants remained stable at 25 µM (49.83 ± 11.8 µg BE g^−1^ DM) and slightly decreased at 250 µM (39.44 ± 3.87 µg BE g^−1^ DM), representing 1.4- and 1.1-fold increases relative to the control, respectively (Figure 9c). After 48 h of culture, TTC levels increased across all treatments. Control plants exhibited a TTC content of 34.6 ± 0.26 µg BE g^−1^ DM. In contrast, H_2_O_2_-treated plants showed higher TTC levels, with a 1.8-fold increase at 25 µM (61.89 ± 0.63 µg BE g^−1^ DM) and a 2.1-fold increase at 250 µM (69.61 ± 0.86 µg BE g^−1^ DM) (Figure 9d).

Due to the extraction procedure and the LC–MS system’s detection limits, compound-level resolution of terpene constituents was limited. Therefore, the presence of terpenes was further corroborated by HPTLC analysis (Supplementary Data S8), using the standards bacchofertin, oleanolic acid, and kingidiol. Chromatographic profiles confirmed the presence of these terpene-related compounds in the extracts.

## 3. Discussion

*Baccharis conferta* synthesizes two main classes of compounds: phenolic and terpene-like metabolites. Each group includes representative molecules, such as flavonoids and neoclerodane-type diterpenes, respectively [3]. In this study, treating *B. conferta* plants with hydrogen peroxide (H_2_O_2_) at two concentrations (25 µM and 250 µM) resulted in significant changes in its specialized metabolism, indicating that H_2_O_2_ acts as a signaling molecule that influences both phenolic and terpene biosynthesis. Evaluating physiological and oxidative stress markers was crucial for distinguishing between controlled elicitation and H_2_O_2_-induced oxidative damage. Although hydrogen peroxide is a ROS, its signaling function depends on its concentration and exposure duration. In this study, no severe chlorosis or necrosis was observed (Figure S1), as confirmed by chlorophyll content measurements that showed no significant differences between H_2_O_2_-treated plants and controls (Table 1). Notably, catalase (CAT) and peroxidase (POD) activities were modulated following H_2_O_2_ treatment, suggesting activation of the antioxidant defense system (Figure 1 and Figure 2). The increased activity of these enzymes indicates an adaptive redox response that helps maintain cellular homeostasis while enabling ROS-mediated signaling.

The effect of H_2_O_2_ on phenolic compounds biosynthesis was evident from increases in total phenolic content (TPC), total flavonoid content (TFC), and PAL activity, 48 h after elicitor application. These variables steadily increased with H_2_O_2_ exposure, reaching their highest levels toward the end of the culture period (Figure 3, Figure 4 and Figure 5). This trend is consistent with previous reports in other species, in which H_2_O_2_ treatment enhanced phenolic accumulation, as seen in *Lens culinaris* [11], *Cicer arietinum* [24], and *Lactuca sativa* [13], *Ficus deltoidea* [25], among others [18]. In *B. conferta*, in addition to increasing phenolic content, elicitation also induced PAL activity. Świeca [11,12] demonstrated that H_2_O_2_ elicitation in *Lactuca sativa* and *Chenopodium quinoa* induced the activity of tyrosine and phenylalanine ammonia-lyases, key enzymes involved in the biosynthesis of polyphenolic compounds. Similarly, in *Ficus deltoidea*, H_2_O_2_ treatment did not affect chlorophyll content, but significantly increased TPC and TFC [25], and in *C. tenuiflora*, H_2_O_2_ exposure was associated with enhanced antioxidant enzyme activity, followed by increased accumulation of phenolic and terpenoid compounds [18].

There is limited information on the specific phenolic compounds produced by *B. conferta* under in vitro conditions. In this study, four flavonoids: flavonoid-I, flavonoid-II, flavonoid-III, and vicenin-2, and five phenolic acids: two caffeoylquinic acids (including chlorogenic acid), and three di-*O*-caffeoylquinic acids were identified in *B. conferta*, and their levels increased in response to H_2_O_2_ exposure. Except for vicenin, these compounds were strongly affected by the elicitation treatments, exhibiting marked and statistically significant changes in their accumulation patterns across the evaluated conditions (Figure 6). Leyva-Peralta [4] reported that *B. conferta* grown under in vitro conditions exhibits high concentrations of caffeoylquinic acids and chlorogenic acid; however, this is the first report of flavonoid-I, flavonoid-II, flavonoid-III, and vicenin-2 in this culture system. Taken together, phenolic acids and flavonoids are phenolic compounds with high antioxidant capacity and play key roles in plant defense mechanisms. Chlorogenic acid production increased over time at both H_2_O_2_ concentrations, with levels rising in both treatments (25 µM and 250 µM) (Figure 7). This pattern has also been described in *Nepeta* species and *Lactuca sativa*, where H_2_O_2_ elicitation led to increases in chlorogenic acid of 17% and 49%, respectively, compared with the control [13,19]. These findings suggest that H_2_O_2_ acts not only as an oxidative stressor but also as a signaling molecule that modulates phenolic metabolism in *B. conferta*. By its nature, H_2_O_2_ increases ROS levels, suggesting that the observed increase in chlorogenic acid may represent a protective response to counteract ROS-induced damage. Chlorogenic acid is known to protect plant cells by reducing membrane lipid peroxidation and scavenging excess ROS [26]. Accordingly, the increased accumulation of phenolic compounds may reflect an adaptive antioxidant response that contributes to redox homeostasis and protection against elicitation-induced oxidative stress.

Furthermore, the effect of H_2_O_2_ on the biosynthesis of terpene-type compounds varied with elicitor concentration. Both treatments increased total terpene content (TTC); however, the lower concentration (25 µM) enhanced *Bco-DXS1* expression, whereas the higher concentration (250 µM) inhibited *Bco-DXS1* expression (Figure 8 and Figure 9). The use of H_2_O_2_ as an elicitor to evaluate terpene production has been minimally explored. In *Castilleja tenuiflora* seedlings, elicitation with 150 µM H_2_O_2_ induced changes in terpene production compared with control plants, as reflected in the relative expression of the *DXS1* gene. In contrast, combined elicitation with salicylic acid (SA) and H_2_O_2_ resulted in higher accumulation of terpene-like compounds, suggesting crosstalk between H_2_O_2_ and SA signaling pathways that enhances their biosynthesis. Similarly, in different *Nepeta* species (*N. nuda* and *N. grandiflora*), exposure to H_2_O_2_ altered terpene composition [19]. In *Pinus massoniana*, *DXS* expression increased after H_2_O_2_ elicitation, likely due to its involvement in stress processes detrimental to the species [27]. Although terpenes are not typically regarded as potent antioxidants against oxidative stress, the observed increase in total terpene content may result from the signaling role of H_2_O_2_, which can modulate metabolic networks regulating the biosynthesis of specialized metabolites [18,19,27]. Despite the limited number of studies evaluating H_2_O_2_ as an elicitor of terpene metabolism, previous reports indicate that it can modulate specific transcription factors (e.g., MYB, WRKY, and MYC) that directly or indirectly regulate genes involved in terpene biosynthesis [10].

**Figure 10:**
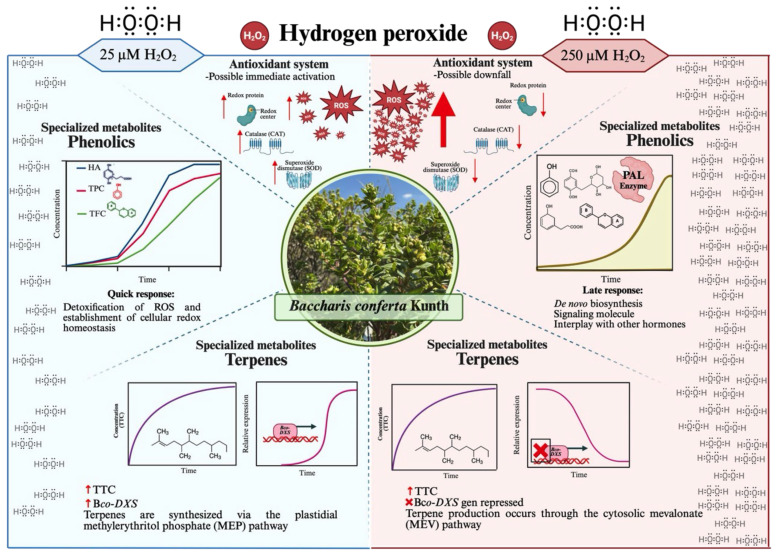
Proposed model summarizing the potential effects of hydrogen peroxide (H_2_O_2_) on *B. conferta* and the biosynthesis of its specialized metabolites. The scheme integrates experimental findings from the present study with previous reports [10,17,25,29] and illustrates a hypothetical regulatory framework linking redox signaling with phenolic and terpene metabolism. At low H_2_O_2_ levels (25 µM; blue-shaded area), there was an increase (**↑**) in total phenolic compounds (**—**), hydroxycinnamic acids (**—**), total flavonoid content (**—**), total terpene content (**—**), and the relative expression of the *Bco-DXS* gene (**—**). Conversely, at a higher H_2_O_2_ concentration (250 µM; red-shaded area), antioxidant activity decreased (**↓**), accompanied by a repression of the *Bco-DXS* gene (**×**). Created in BioRender. Trejo tapia, G. (2026) https://BioRender.com/r2du6np (accessed on 4 February 2026). *Bco*: *Baccharis conferta*; *DXS*: 1-deoxy-D-xylulose-5-phosphate synthase; HA: hydroxycinnamic acids; H_2_O_2_: Hydrogen peroxide; MEcPP: methylerythritol cyclodiphosphate; PAL: Phenylalanine ammonia-lyase; ROS: Reactive Oxygen Species; TFC: Total flavonoid content; TPC: Total phenolic content; TTC: Total terpene content.

The results of this study suggest that the biosynthesis of specialized metabolites in *B. conferta* is differentially modulated by both exposure time and H_2_O_2_ concentration. Exposure to H_2_O_2_ exerts a dose-dependent regulatory effect on the specialized metabolic pathways of *B. conferta* over time (Figure 10). This behavior can be attributed to the plant’s intrinsic defense mechanisms, its capacity for de novo synthesis of specialized metabolites, and the signaling role of H_2_O_2_, which actively participates in regulating plant defense responses [8]. Although H_2_O_2_ is generally regarded as a toxic molecule, its use as a plant elicitor is now well recognized due to its dual function. At high concentrations, H_2_O_2_ can cause irreversible cellular damage; however, at low concentrations, it acts as a signaling molecule that activates various antioxidant and defense-related pathways. In both scenarios, H_2_O_2_ triggers defense mechanisms that enhance the biosynthesis and activation of specialized metabolites [9,13]. The biosynthesis of specialized metabolites is tightly regulated by a network of signaling molecules, ensuring their precise temporal and spatial induction [28]. The immediate increase in specialized metabolites observed at the lower H_2_O_2_ concentration (25 µM) in *B. conferta* may reflect a rapid activation of the plant’s defense system in response to a transient rise in ROS, providing an early protective response. The accumulation of phenolic-like compounds plays a key role in this initial defense phase, and H_2_O_2_ likely contributes through a feedback mechanism that stimulates multiple genes involved in phenolic biosynthesis [13,14,18,29]. Conversely, exposure to a higher H_2_O_2_ concentration (250 µM) may reflect the plant cell’s adaptive plasticity under prolonged or severe stress conditions. Although high levels of H_2_O_2_ can be toxic, they may also induce defense responses associated with ROS detoxification and the re-establishment of cellular redox homeostasis [10,29]. In *B. conferta*, a high ROS scenario could temporarily deplete the antioxidant system, triggering the *de novo* synthesis of specialized metabolites and delaying their activation, as evidenced by the increase in PAL activity observed after elicitation.

The overexpression of *Bco-DXS* at 25 µM H_2_O_2_ and its repression at 250 µM, together with the presence and increase in terpene content, suggest that low H_2_O_2_ concentrations may modulate terpene biosynthesis via the plastidial methylerythritol phosphate (MEP) pathway. However, additional molecular and enzymatic evidence would be needed to validate this proposed mechanism. In *Populus trichocarpa* and other species, recombinant protein studies have shown that the DXS enzyme limits the rate of isoprenoid precursor biosynthesis in the MEP pathway [20]. Conversely, higher H_2_O_2_ concentrations, associated with elevated ROS levels, may repress the MEP pathway, promoting terpene synthesis primarily via the cytosolic mevalonate (MEV) pathway [16,27]. Notably, the MEP pathway has been reported to be particularly sensitive to oxidative stress, in which excessive ROS can lead to the accumulation of intermediates, such as methylerythritol cyclodiphosphate (MEcPP). MEcPP acts as a retrograde signaling molecule, modulating *DXS* gene expression and inducing stress-responsive genes [14]. This mechanism could explain the repression of *Bco*-*DXS* at high H_2_O_2_ concentrations and the sustained terpene production observed under both conditions. Overall, these findings are consistent with a role of H_2_O_2_-mediated redox signaling in influencing *Bco-DXS* expression and terpene biosynthesis in *B. conferta.* Future studies will be essential to determine whether differential regulation occurs between the plastidial (MEP) and cytosolic (MEV) pathways under these conditions.

## 4. Materials and Methods

### 4.1. Chemical Reagents

All reagents were of analytical grade and used as received unless otherwise specified.

For plant culture establishment, Murashige and Skoog (MS) basal medium (Sigma-Aldrich^®^, St. Louis, MO, USA, M5519), Phytagel (Sigma-Aldrich^®^ P8169, St. Louis, MO, USA), and sucrose (Sigma-Aldrich^®^, St. Louis, MO, USA) were employed. Hydrogen peroxide treatments were performed using H_2_O_2_ (Sigma-Aldrich^®^, H1009, St. Louis, MO, USA) prepared with ultra-pure water obtained from a Milli-Q purification system (Merck Millipore, Burlington, MA, USA).

Reagents used for oxidative stress evaluation and antioxidant enzyme assays included disodium hydrogen phosphate (Na_2_HPO_4_, 3822-01, J.T. Baker, Radnor, PA, USA), sodium dihydrogen phosphate (NaH_2_PO_4_, T9159, Sigma-Aldrich^®^, St. Louis, MO, USA), guaiacol (Sigma-Aldrich^®^, G5502, St. Louis, MO, USA), potassium iodide (83842, Fermont, Nuevo León, México), Tris-base (44109, J.T. Baker, Radnor, PA, USA), Tris–HCl (4103-01, J.T. Baker, Radnor, PA, USA), trichloroacetic acid (TCA; Sigma-Aldrich^®^, T9159, St. Louis, MO, USA), Bradford reagent (Sigma-Aldrich^®^, B6916, St. Louis, MO, USA), dithiothreitol (DTT, 1610611, Bio-Rad, Hercules, CA, USA), EDTA disodium salt (04802, Fermont, Nuevo León, México), poly(vinylpyrrolidone) (77727, Fluka Analytical (Merck), Buchs, Switzerland), and L-phenylalanine (Sigma-Aldrich^®^, P2126, St. Louis, MO, USA).

For chemical analyses, aluminum chloride (AlCl_3_; Sigma-Aldrich^®^, 206911, St. Louis, MO, USA), chlorogenic acid (≥98% purity; Sigma-Aldrich^®^, C3878, St. Louis, MO, USA), gallic acid (Sigma-Aldrich^®^, G7384, St. Louis, MO, USA), rutin (Sigma-Aldrich^®^, R-9000, St. Louis, MO, USA), Folin–Ciocalteu reagent (Sigma-Aldrich^®^, 47641, St. Louis, MO, USA), sodium carbonate (Sigma-Aldrich^®^, S5506, St. Louis, MO, USA), sodium nitrate (Sigma-Aldrich^®^, 223530, St. Louis, MO, USA), sodium hydroxide (J.T. Baker, Radnor, PA, USA, B5947-05), sulfuric acid (Fermont, Nuevo León, México, 01615), chloroform (J.T. Baker, Radnor, PA, USA, 9180-03), dichloromethane (Fermont, Nuevo León, México, 06235), methanol (J.T. Baker, Radnor, PA, USA, 9070-03), methanol HPLC grade (J.T. Baker, Radnor, PA, USA, 9093-03), and formic acid (J.T. Baker, Radnor, PA, USA, 012802) were used.

Molecular analyses were conducted using Phusion High-Fidelity DNA Polymerase (Thermo Scientific™, Waltham, MA, USA, F530S), the pJET1.2 cloning vector (Thermo Scientific™, Waltham, MA, USA, K1232), PureLink Plant RNA Reagent (Thermo Scientific™, Waltham, MA, USA, 12322012), TURBO DNase (Invitrogen™ Waltham, MA, USA, AM1907), the RevertAid First Strand cDNA Synthesis Kit (Thermo Scientific™, Waltham, MA, USA, K1621), and SYBR Green PCR Master Mix (Applied Biosystems^®^ Waltham, MA, USA, QR0100).

### 4.2. Plant Material

Shoot cultures of *Baccharis conferta* were established and propagated in vitro as previously described [4]. For the experiment, four-week-old in vitro shoots were used. Three uniform nodal segments (2 cm) were placed in Magenta boxes containing semi-solid MS medium [30], supplemented with 30 g L^−1^ sucrose and 2.2 g L^−1^ Phytagel adjusted to pH 5.8. The plants were maintained at 25 ± 2 °C under a 16 h light/8 h dark photoperiod with a light intensity of 77 µmol m^−2^ s^−1^.

### 4.3. H_2_O_2_ Treatments

To evaluate the effect of exogenous hydrogen peroxide application on *B. conferta* plants, thirty-day-old plants from the initial culture were used. Elicitation was performed by foliar spraying. H_2_O_2_ was applied at 25 µM and 250 µM. Samples were collected at four specific times: 0 h (baseline), 9 h, 24 h, and 48 h post-treatment. Control plants, sprayed with distilled water, were collected at the same time points. To ensure sufficient plant material for all determinations, each treatment and time point consisted of three Magenta boxes, each containing three plants. Each box was considered an independent experimental unit. The number of biological replicates analyzed for each determination is indicated in the corresponding graphic.

### 4.4. Assessment of Physiological and Oxidative Stress Responses

#### 4.4.1. Chlorophyll Content

Relative chlorophyll content in leaves was measured with a SPAD-502 chlorophyll meter (Konica Minolta, Tokyo, Japan). Visible stress symptoms were monitored and documented photographically. Chlorophyll values were expressed as µg cm^−2^.

#### 4.4.2. Antioxidant Enzyme Activity: Catalase (CAT) and Peroxidase (POD)

Fresh tissue (0.5 g) was used for enzyme extraction. Samples were ground in liquid nitrogen and homogenized in 50 mM Tris–HCl buffer (pH 7.5). Soluble protein content was quantified using the Bradford reagent [31]. CAT activity was measured spectrophotometrically at 240 nm. The reaction mixture contained 50 mM Tris buffer (pH 7.0), 5 mM H_2_O_2_. POD activity was measured at 425 nm using guaiacol as substrate. The reaction mixture contained 100 mM Tris buffer (pH 7.0), 5 mM H_2_O_2_, guaiacol, and enzyme extract. The molar extinction coefficients used were 39.4 mM^−1^ cm^−1^ and 26.6 mM^−1^ cm^−1^ for CAT and POD, respectively. All measurements were performed in triplicate, with n = 3, and enzyme activities were expressed as µmol min^−1^ mg^−1^ protein [18].

### 4.5. Phenylalanine Ammonia Lyase (PAL) Activity

Phenylalanine ammonia-lyase (PAL) activity was measured by monitoring cinnamic acid production from phenylalanine at 290 nm. The results were expressed as nmol cinnamic acid h^−1^ µg^−1^ protein. Enzyme extracts were obtained from frozen plant material that had been ground in liquid nitrogen. Protein content was quantified using the Bradford method with a NanoDrop spectrophotometer (NanoDrop 2000; Thermo Scientific™, Waltham, MA, USA), following the manufacturer’s instructions [18,31].

### 4.6. Preparation of Extracts for Chemical Analysis

Plant material from elicited and non-elicited plants was harvested and dried in a convection oven (StableTemp; Cole-Parmer, Vernon Hills, IL, USA) at 50 °C for 48 h to constant weight. The dried tissue was ground into a fine powder and extracted by maceration with dichloromethane, followed by methanol. Extraction was performed at room temperature for 48 h at a 1:10 weight-to-volume ratio. The extracts were filtered through Whatman No. 1 filter paper, and the solvents were removed under reduced pressure at 40 °C. This process was repeated three times for each sample. Finally, the extracts were lyophilized and stored at −20 °C in the dark until analysis [3]. The methanolic extract was used for the analysis of phenolic compounds: total phenolic compounds (TPC), total flavonoids (TFC), and LC-MS analysis. The dichloromethane extract was used for terpene analysis by HPTLC-based terpene profiling and for total terpene content (TTC).

### 4.7. Quantification of Total Phenolic Compounds (TPC), Flavonoids (TFC), and Total Terpenes (TTC)

The quantification of total phenolic compound (TPC) and total flavonoid (TFC) contents was performed according to the method reported by Nazir [10]. The quantification of total terpene content (TTC) was performed according to the procedure described by Aloisio [32]. All three techniques were applied with certain modifications. A UV–Visible spectrophotometer (Shimadzu UV-1800, Kyoto, Japan) was used to measure absorbance, using 1 mL quartz cuvettes for each reaction.

#### 4.7.1. Quantification of Phenolic Compounds (TPC)

The Folin–Ciocalteu method was used to quantify TPC. A total of 100 μL of extract was diluted with 500 μL of distilled H_2_O, and 100 μL of Folin–Ciocalteu reagent was added. The mixture was incubated for 6 min. Subsequently, 1 mL of 7% Na_2_CO_3_ and 500 μL of distilled H_2_O were added, and the mixture was left to stand for 90 min. Absorbance was measured at 750 nm. The standard calibration curve was prepared using gallic acid: y = 0.0865x − 0.0094; R^2^ = 0.999. Results are expressed as gallic acid equivalents per gram of dry matter (µg GAE g^−1^ DM).

#### 4.7.2. Quantification of Total Flavonoid Content (TFC)

Total flavonoid content (TFC) was measured using the AlCl_3_ colorimetric method. Samples were diluted with distilled H_2_O (1:5), and 100 μL of 5% NaNO_3_ was added, followed by incubation for 6 min. Subsequently, 150 μL of 10% AlCl_3_ was added, and the mixture was allowed to stand for 5 min. The reaction was stopped by adding 200 μL of 1 M NaOH. Absorbance was measured at 510 nm. The standard calibration curve was prepared using rutin: y = 0.0103x + 0.0467; R^2^ = 0.975. TFC was expressed as rutin equivalents per gram of dry matter (µg RE g^−1^ DM).

#### 4.7.3. Quantification of Total Terpene Content (TTC)

Total terpene content (TTC) was measured spectrophotometrically at 538 nm. A mixture of 200 μL of the dichloromethane extract and 1.5 mL of chloroform was prepared and allowed to stand for 3 min. Then, 100 μL of sulfuric acid was added, and the mixture was incubated in the dark for 1 h. The supernatant was carefully discarded, and the precipitate was vigorously mixed with methanol. Absorbance was recorded at 538 nm. The standard calibration curve was prepared using Bacchofertin (reference standard obtained according to the method described previously [3]); y = 0.0003x − 0.0074; R^2^ = 0.9956. Results were expressed as Bacchofertin equivalents per gram of dry matter (µg BE g^−1^ DM).

### 4.8. Chromatography Analysis

#### 4.8.1. LC Analysis (Liquid Chromatography) and Quantification of Hydroxycinnamic Acids

Chromatographic separation and spectrometric analysis were performed using an LC-MS/2020 system (Shimadzu, Tokyo, Japan). The system consisted of a CBM-20A system controller, two LC-20AD binary pumps, a DGU-20A5R degasser, a SIL-20AC autosampler, a CTO-20A column oven, an SPD-M20A UV–Vis photodiode array detector, and a single-quadrupole mass spectrometer (LCMS-2020) equipped with an electrospray ionization (ESI) source. Data acquisition and processing were carried out using LCMS Solutions v5.0 software. Samples were eluted and analyzed at 30 °C using a reversed-phase Lichrospher^®^ RP-18 column (100 mm × 250 mm × 4 mm, 5 µm; Merck, Darmstadt, Germany). The mobile phase consisted of 0.2% formic acid aqueous solution as solvent A and 0.2% formic acid in methanol solution as solvent B at 1.0 mL min^−1^. Gradient elution was conducted as follows: 0–1 min, 70–30% A-B; 1–3 min, 67–33% A-B; 3–7 min, 63–37% A-B; 7–10 min, 60–40% A-B; 10–13 min, 50–50% A-B; 13–16 min, 45–55%, A-B; 16–22 min, 40–60% A-B; 22–25 min, 55–45%, A-B; 25–27 min, 70–30%, A-B [33].

Compounds were identified according to their retention times [Rt (min)], deprotonated molecular ions observed in negative ESI mode ([M–H]^−^, *m*/*z*), and UV absorption spectra (λmax at 325 nm). Tentative identification was supported by comparison with literature data. Each peak detected in the chromatogram is described in Supplementary Data S2. The relative abundance of compounds (Flavonoid I, II, and III, Caffeoylquinic acid, Vicenin-2, and di-*O*-caffeoylquinic acid I, II, and III) was estimated from the area of each peak and its contribution to the total signal area.

Relative abundance (RA) was determined as the percentage of each peak area relative to the total chromatographic area per sample. RA values were log10-transformed to reduce heteroscedasticity and to approximate normality. To avoid undefined log10(0) values, a pseudocount of 1 was added to all observations before transformation. The resulting log-transformed values (RA^log10^) were used for statistical comparisons among treatments. Metabolites with a *p*-value < 0.05 and a fold change (FC) ≥ 1.5 or FC ≤ 0.5 will be considered differentially accumulating metabolites (DAM) [34,35].

Chlorogenic acid was quantified using an external calibration curve prepared with an authentic standard. Standard solutions were prepared at concentrations ranging from 3 to 500 µg mL^−1^. The calibration curve was constructed by plotting peak area versus concentration (y = 43,289x; R^2^ = 0.9959). Results are expressed as µg g^−1^ dry weight (DW). Quantification was performed using three independent biological replicates. The calibration curve is shown in Table S4 and Figure S4 (Supplementary Data S4).

#### 4.8.2. High Performance Thin-Layer Chromatography (HPTLC)

HPTLC was performed on normal-phase TLC silica gel 60 F254 plates (20 × 10 cm; Merck, Germany) with dichloromethane/methanol (95:5, *v*/*v*) as the mobile phase. Dichloromethane extracts and reference standards (oleanolic acid, bacchofertin, bacchofertone, and a kingidiol fraction) were prepared at 8 mg mL^−1^ in methanol. 10 µL of each sample was automatically applied using a CAMAG Linomat V (Muttenz, Switzerland). The samples were applied with a width of 6 mm and positioned 8 mm from the lower edge of the plate. The chromatographic plates were developed in a chamber previously saturated with the mobile phase of dichloromethane/methanol (95:5, *v*/*v*) to a migration distance of 80 mm (solvent front). The bands were visualized under UV light at 254 and 366 nm. Subsequently, Komarowsky (KOM) reagent was applied, and the plates were examined under visible light (540 nm). Finally, the plates were scanned and analyzed using VisionCATS software (version 2.4.17207.2).

### 4.9. Isolation, Cloning, and Bioinformatic Analysis of the Partial Sequence of the DXS Gene from B. conferta

The partial sequence of the 1-deoxy-*D*-xylulose-5-phosphate synthase 1 (*Bco-DXS*) gene was isolated using a pair of degenerate oligonucleotides. Amplification, cloning, and bioinformatic analysis of the sequence were performed as previously described by Rubio-Rodríguez [18].

The oligonucleotides were designed based on conserved regions of sequences phylogenetically related to *B. conferta*. Two µL of cDNA and degenerate primers (100 µM) were used together with the components of the Phusion High-Fidelity DNA Polymerase (F530S; Thermo Scientific™) in a final reaction volume of 25 µL. The amplified product was cloned into the pJET1.2 vector (K1232; Thermo Scientific™) by electroporation (1 mm, 165,208; 1.8 kV, 25 µF, 200 Ω, Gene Pulser Xcell™ Electroporation System; Bio-Rad). The cloned product was sequenced at the DNA Synthesis and Sequencing Unit (Instituto de Biotecnología, Universidad Nacional Autónoma de México; Morelos, México).

The reported DXS sequences (isoforms I, II, and III) for the taxon Embryophyta were retrieved from NCBI for the in silico sequence analysis. Bioinformatic analyses were performed using online software tools: BioEdit Sequence Alignment Editor (version 7.1.1) (open reading frames [ORFs], in silico translation of the amino acid sequence, alignment [ClustalW], comparison, editing, and visualization), PhyML v3.1/3.0 aLRT (phylogenetic tree construction using the maximum likelihood method), and MUSCLE (alignment; bootstrap analysis with 1000 replicates for branch support, internal branch reliability assessed using the aLRT test).

### 4.10. Expression Analysis of Bco-DXS

Total RNA from elicited and non-elicited *B. conferta* plants was isolated using the PureLink™ Plant RNA Reagent, following the manufacturer’s instructions. RNA was quantified using a NanoDrop spectrophotometer (NanoDrop 2000; Thermo Scientific™), and its integrity was assessed by denaturing agarose gel electrophoresis. To remove residual genomic DNA, DNase was used according to the manufacturer’s protocol. First-strand cDNA synthesis was carried out using the RevertAid First Strand cDNA Synthesis Kit, following the manufacturer’s guidelines.

Specific primers for the partial *Bco-DXS* sequence were designed using the online software Primer3Plus (https://www.primer3plus.com/index.html accessed on 29 August 2023). Quantitative real-time PCR (qRT-PCR) assays were performed with SYBR Green PCR Master Mix in a final reaction volume of 20 µL, using 10 µM of each primer. Amplification reactions were conducted on a StepOne Real-Time PCR System with StepOne Software v2.1 (Applied Biosystems). Gene expression levels were analyzed using the Pfaffl method [36]. The elongation factor 1 (*EF1*) gene was used as the reference housekeeping gene.

The thermocycler conditions were: 15 min at 95 °C for activation, followed by 40 PCR cycles: 15 s at 95 °C for denaturation, 15 s at 60 °C for annealing, and 30 s at 72 °C for extension. Melting curve conditions were 15 s at 95 °C for denaturation, 15 s at 60 °C for annealing, and 15 s at 95 °C for denaturation, with a 0.3 °C increase per cycle. The oligonucleotides used are: *Bco-DXS*: F: 5′-AGG CAG GAT TTC TTG GTG CA-3′ and R: 5′-AGT TTA CGG AAT TCG GGG CT-3′ [Efficiency: 1.98]. *Bco-EF1*: F: 5′-CTG AAG TTA AGT CTG TTG AGA TGC ACC-3′ and R: 5′-GCC AGG GTG GTT CAT GAT GAT GAC C-3′ [*Efficiency*: 1.86].

### 4.11. Statistical Analysis

Statistical analyses were performed using GraphPad Prism version 10.5.0 for macOS (Systat Software; GraphPad Software, www.graphpad.com, Boston, MA, USA). Data are presented as mean ± standard error. To assess the statistical significance of differences between treatments, one-way or two-way analysis of variance (ANOVA) was followed by Tukey’s and Dunnett’s multiple comparison tests. The normality of the raw data was confirmed using the D’Agostino-Pearson statistical test. *p*-values less than 0.05 (*p* ≤ 0.05) were considered significantly different. Statistical metrics are provided in the corresponding figures.

## 5. Conclusions

Hydrogen peroxide functions as an oxidative signaling molecule, promoting the biosynthesis of specialized metabolites in *B. conferta*. This study demonstrates that external H_2_O_2_ acts as a signaling agent regulating phenylpropanoid and terpenoid biosynthesis in the plant. The responses were clearly dose-dependent and showed dynamic changes over time, with a distinct separation between elicitation and oxidative damage effects. A low H_2_O_2_ level (25 µM) rapidly triggered strong activation of phenylalanine ammonia-lyase (PAL), boosting metabolic flux through the phenylpropanoid pathway. This response showed significant increases in TFC and TPC, along with the accumulation of chlorogenic acid and caffeoylquinic acid derivatives. At the same time, H_2_O_2_ influenced terpene metabolism by selectively regulating *Bco-DXS1*, a critical enzyme in the MEP pathway. Lower H_2_O_2_ levels increased *Bco-DXS1* expression and terpene production, whereas higher concentrations partially suppressed gene activity while still maintaining terpene levels, suggesting possible post-transcriptional regulation or metabolic compensation. Overall, the findings support the use of controlled H_2_O_2_ elicitation as a practical approach to boost the production of specialized metabolites in in vitro cultures without harming plant health.

## Figures and Tables

**Table 1:** Physiological response of *Baccharis conferta* plants to hydrogen peroxide (H_2_O_2_) elicitation: fresh biomass and chlorophyll content.

	Fresh Biomass (g)			Chlorophyll Content (µg cm^−2^)		
	C	25 µM	250 µM	C	25 µM	250 µM
0 h	1.06 ± 0.4 _a_	1.06 ± 0.4 _a_	1.06 ± 0.4 _a_	26.62 ± 1.6 _a_	26.62 ± 1.6 _a_	26.62 ± 1.6 _a_
9 h	0.79 ± 0.3 _a_	1.03 ± 0.8 _a_	0.77 ± 0.7 _a_	26.38 ± 1.2 _a_	28.83 ± 1.0 _a_	26.26 ± 2.4 _a_
24 h	0.79 ± 0.3 _a_	1.32 ± 1.5 _a_	0.78 ± 1.3 _a_	26.50 ± 0.4 _a_	27.40 ± 3.4 _a_	23.94 ± 0.8 _a_
48 h	0.88 ± 0.6 _a_	1.35 ± 1.0 _a_	1.38 ± 1.2 _a_	26.50 ± 0.4 _a_	25.50 ± 3.1 _a_	25.52 ± 0.9 _a_

Data are presented as mean ± standard error (SE) of three biological replicates (n = 3). Statistical analyses were performed independently for each time point using one-way analysis of variance (ANOVA), followed by Tukey’s multiple comparison test to determine significant differences among treatments (*p* ≤ 0.05). Within each row, values followed by different letters are significantly different among treatments. Treatments were defined as: C (control), 25 µM, and 250 µM H_2_O_2_.

## Data Availability

The original contributions presented in this study are included in the article/Supplementary Material. The information obtained from the *BcoDXS* sequence has been deposited in the NCBI database and registered under the ID OP047919. Further inquiries can be directed to the corresponding authors.

## Supplementary Materials

The following supporting information can be downloaded at: https://www.mdpi.com/article/10.3390/ijms27062544/s1.

## Abbreviations

The following abbreviations are used in this manuscript:

*Bco*	*Baccharis conferta*
BE	Bacchofertin Equivalents
CAT	Catalase
*DXS*	1-deoxy-D-xylulose-5-phosphate synthase
DAM	Differentially accumulating metabolites
DW	Dry weight
*EF1*	Elongation factor 1
GAE	Gallic Acid Equivalents
HA	Hydroxycinnamic acids
HPTLC	High-Performance Thin-Layer Chromatography
H_2_O_2_	Hydrogen peroxide
LC-MS	Liquid chromatography-tandem mass spectrometry
PAL	Phenylalanine ammonia-lyase
POD	Peroxidase
RA	Relative abundance
RA^log10^	Log10-transformed relative abundance
RE	Rutin Equivalents
ROS	Reactive Oxygen Species
TFC	Total flavonoid content
TPC	Total phenolic compounds
TTC	Total terpene content
MEcPP	methylerythritol cyclodiphosphate
MEP	Methylerythritol 4-phosphate pathway
MEV	Mevalonate pathway
