# Supplementary material for: Hydrogen Peroxide-Oxidative Signaling Enhances Biosynthesis of Specialized Metabolites in Baccharis conferta Kunth
Source: Int J Mol Sci. 2026 Mar 10;27(6):2544. doi: 10.3390/ijms27062544 (PMC13027281; doi:10.3390/ijms27062544)
Supplement: Supplementary file 1 [file ijms-27-02544-s001.zip › Supplementary Data S4. LC–MS Conditions for the Calibration Curve of Chlorogenic Acid.pdf]

Supplementary Data 4. LC–MS Conditions for the Calibration Curve of Chlorogenic Acid

Table S4. Standard curve data for chlorogenic acid, showing peak areas at different concentrations at 325 nm.

Concentration (μg/mL)	Absorbance			
	Replicate-I	Replicate-II	Replicate-III	Mean
3.9	197746	197646	196615	197336
15.62	800609	799110	802703	800807
62.5	3183819	3198061	3203426	3195102
250	12100207	12091659	12127411	12106426
500	20989450	20945374	20859797	20931540

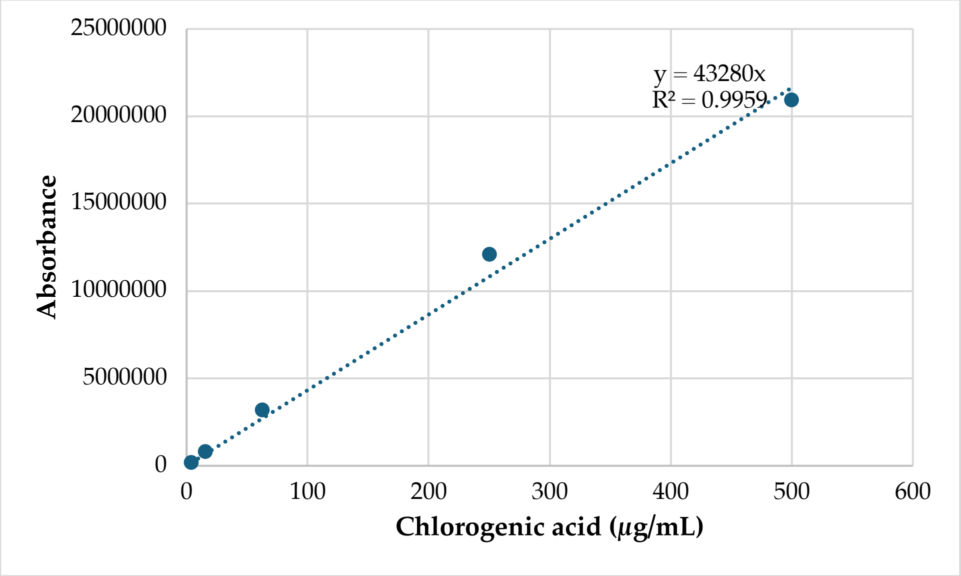

Figure S4. Calibration curve of Chlorogenic Acid

The analytical method was developed at the Chromatography Laboratory of the Department of Biotechnology, Centro de Desarrollo de Productos Bióticos (CEPROBI-IPN), by M.Sc. Virginia Medina Pérez.
Standard: Chlorogenic acid (C3878; Sigma-Aldrich, ≥98% purity)
Instrumentation. Analyses were performed using an LCMS-2020 system (Shimadzu, Tokyo, Japan).
Sample. Powdered analytical standards were used to prepare the calibration curve.
Chromatographic Conditions

- Column: Reverse-phase RP-18 column (Lichrospher 100, 250 × 4 mm, RP-18, 5 μm; Merck, Darmstadt, Germany) connected to a guard column.
- Injection volume: 20 μL
- Flow rate: 1 mL min^{-1}
- Column oven temperature: 30°C
- Run time: 27 min

Mobile Phases. All solvents were HPLC grade

- Solvent A: Milli-Q water with 0.2% formic acid
- Solvent B: Methanol with 0.2% formic acid

Gradient Program
0.01–1 min: 30% B
1–3 min: 33% B

3–7 min: 37% B
7–10 min: 40% B
10–13 min: 50% B
13–16 min: 55% B
16–22 min: 60% B
22–25 min: 45% B
25–27 min: 30% B
Detection. UV detection wavelength: 325 nm
LC Modules

- System controller: CBM-20A
- Binary pumps: 2 × LC-20AD
- Degasser: DGU-20A5R
- Autosampler: SIL-20AC
- Column oven: CTO-20A
- Photodiode array detector (UV-Vis): SPD-M20A

Mass Spectrometry Conditions

- Mass spectrometer: LCMS-2020 single quadrupole
- Ionization source: Electrospray ionization (ESI)
- Ionization mode: Negative and positive
- Dry gas (N_2): 10 L min^{-1}
- Nebulizing gas flow: 1.54 L/min
- Interface voltage: 4.5 kV
- Detector voltage: 1.2 kV
- Scan range: m/z 50–1000 and 400–900

Software. Data acquisition and processing were performed using LabSolutions software (version 5.0).
