# Supplementary material for: Hydrogen Peroxide-Oxidative Signaling Enhances Biosynthesis of Specialized Metabolites in Baccharis conferta Kunth
Source: Int J Mol Sci. 2026 Mar 10;27(6):2544. doi: 10.3390/ijms27062544 (PMC13027281; doi:10.3390/ijms27062544)
Supplement: Supplementary file 1 [file ijms-27-02544-s001.zip › Supplementary Data S1. Morphological responses of Baccharis conferta plants.pdf]

Supplementary Data S1. Morphological responses of *Baccharis conferta* plants.

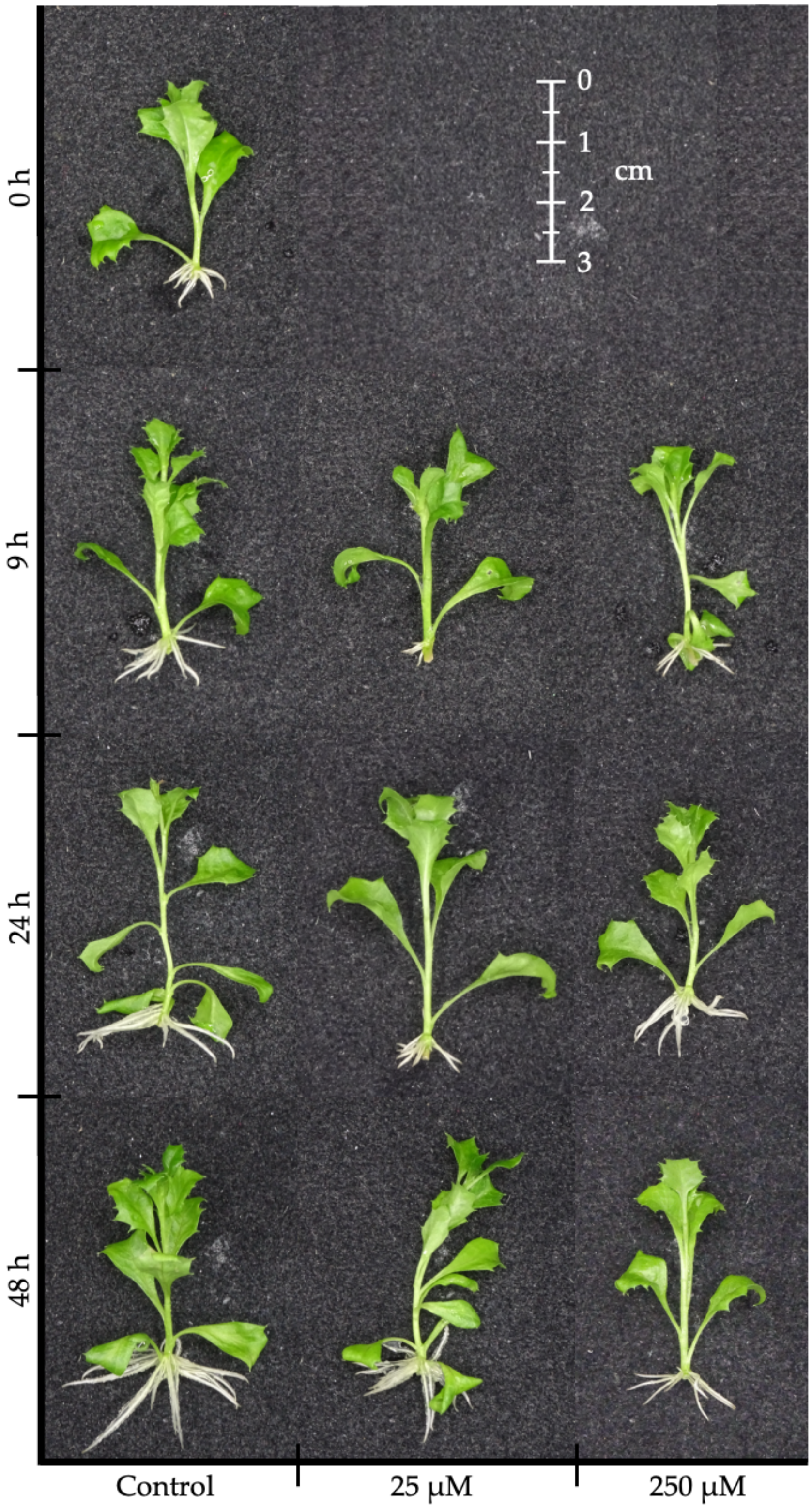

Figure S1. Morphological responses of *Baccharis conferta* plants to hydrogen peroxide (H_2O_2) elicitation. Representative plants from the control group and those treated with 25 and 250 µM H_2O_2 were evaluated at 0, 9, 24, and 48 hours after elicitation.
