# Supplementary material for: Hydrogen Peroxide-Oxidative Signaling Enhances Biosynthesis of Specialized Metabolites in Baccharis conferta Kunth
Source: Int J Mol Sci. 2026 Mar 10;27(6):2544. doi: 10.3390/ijms27062544 (PMC13027281; doi:10.3390/ijms27062544)
Supplement: Supplementary file 1 [file ijms-27-02544-s001.zip › Supplementary Data S2. Chemical compounds identified in B. conferta by LC-PDA-ESI-MS.pdf]

Supplementary Data S2. Chemical compounds identified in *B. conferta* by LC-PDA-ESI-MS.

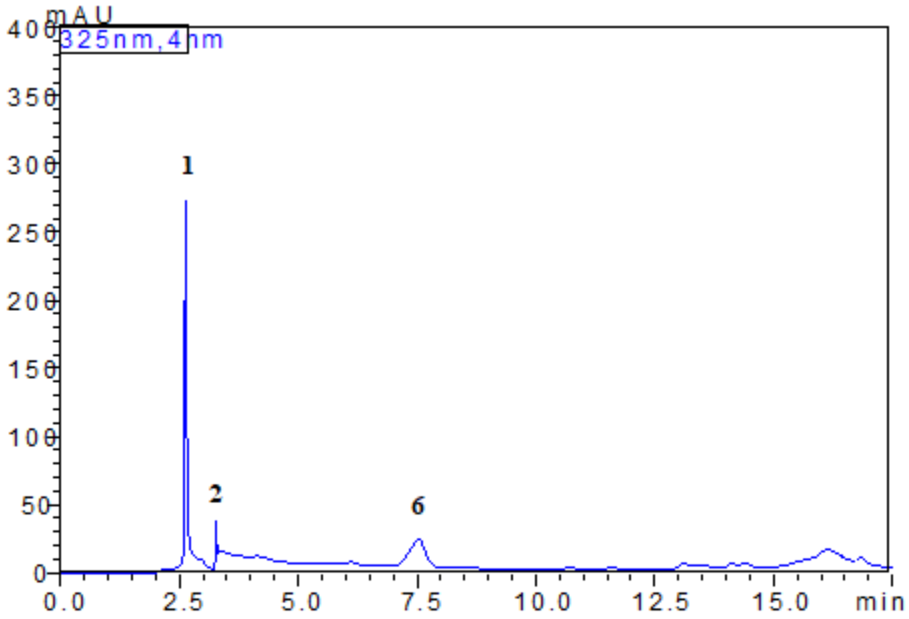

Figure S2-1. LC-MS profile of *B. conferta* plants without elicitation (control) at 325 nm.

Table S2. Compounds identified in *B. conferta* extracts by LC-MS at 325 nm.

Peak	Rt (min)	Ion [M-H]- (m/z)	Class	Name	Compound	UV max
1	2.62	293	Flavonoid	Flavonoid-I	Unidentified	223, 237, 271
2	3.33	257		Flavonoid-II	Unidentified	223, 259, 274
3	4.40	353		Flavonoid-III	Unidentified	213, 297, 323
4	5.77	353	Caffeoylquinic acid	Caffeoylquinic acid	Unidentified	213, 324
5	6.21	353		Chlorogenic acid	Chlorogenic acid	214, 326
6	7.66	593	Flavonoid	Vicenin-2	Vicenin-2	213, 271, 330
7	11.93	515	di-*O*-caffeoylquinic acid	di-*O*-caffeoylquinic acid-I	Unidentified	213, 324
8	13.07	515		di-*O*-caffeoylquinic acid-II	Unidentified	216, 328
9	14.40	515		di-*O*-caffeoylquinic acid-III	Unidentified	215, 326

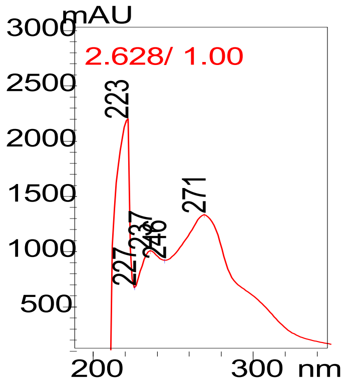

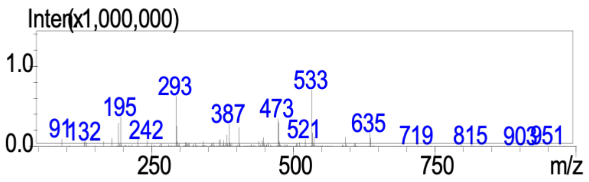

Figure S2-2. UV spectra and mass spectra of compound **1** (Rt=2.62 min) from *B. conferta*.

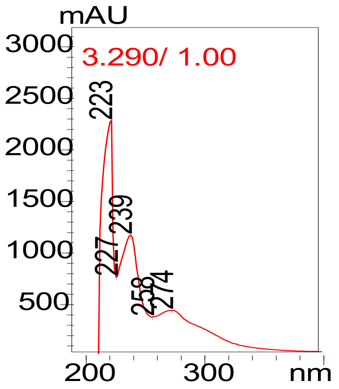

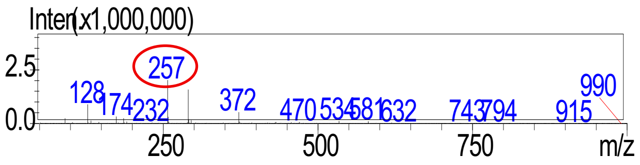

Figure S2-3. UV spectra and mass spectra of compound **2** (Rt=3.33 min) from *B. conferta*.

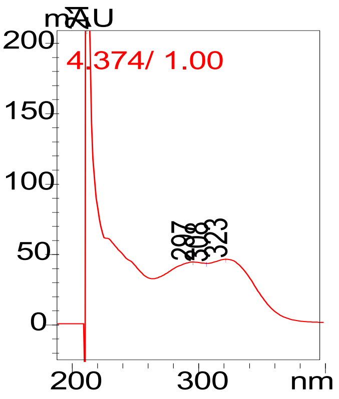

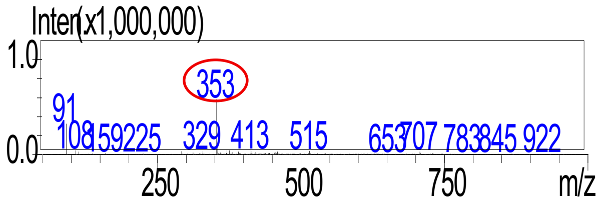

Figure S2-4. UV spectra and mass spectra of compound **3** (Rt=4.40 min) from *B. conferta*.

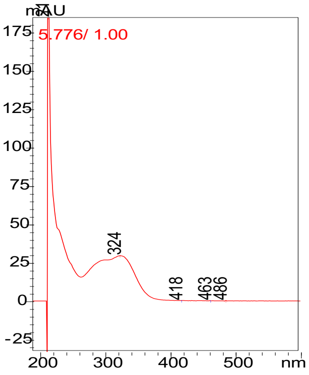

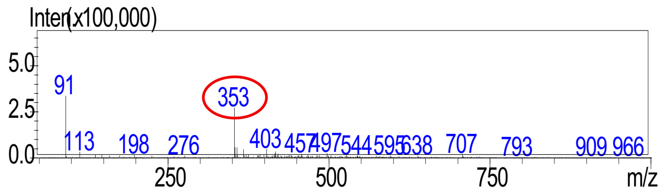

Figure S2-5. UV spectra and mass spectra of compound **4** (Rt=5.77 min) from *B. conferta*.

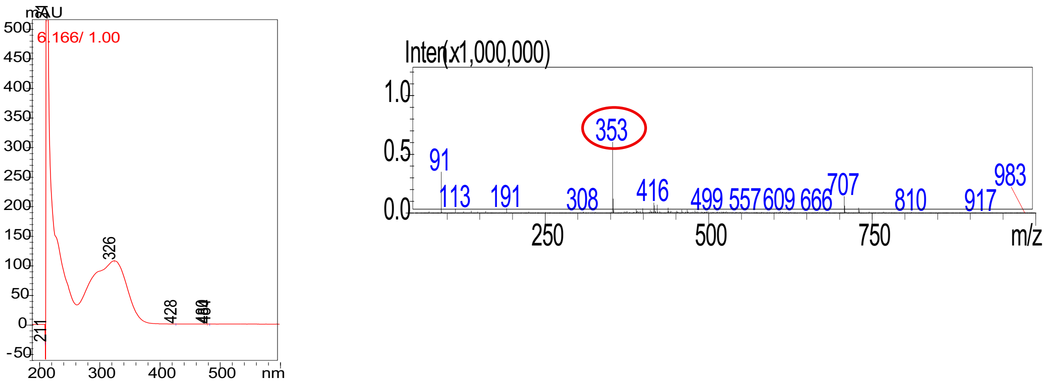

Figure S2-6. UV spectra and mass spectra of compound **5** (Rt=6.21 min) from *B. conferta*.

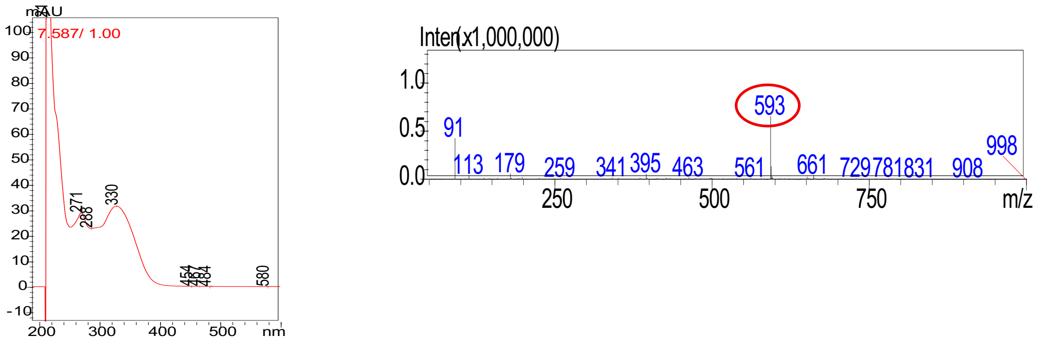

Figure S2-7. UV spectra and mass spectra of compound **6** (Rt=7.66 min) from *B. conferta*.

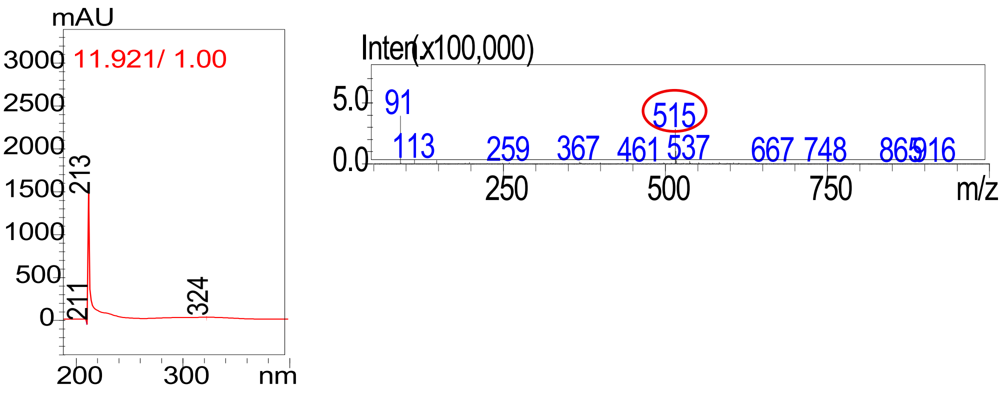

Figure S2-8. UV spectra and mass spectra of compound **7** (Rt=11.93 min) from *B. conferta*.

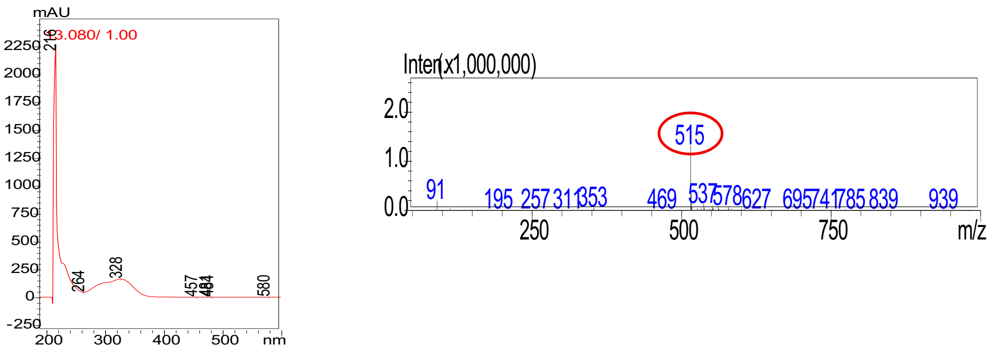

Figure S2-9. UV spectra and mass spectra of compound **8** (Rt=13.07 min) from *B. conferta*.

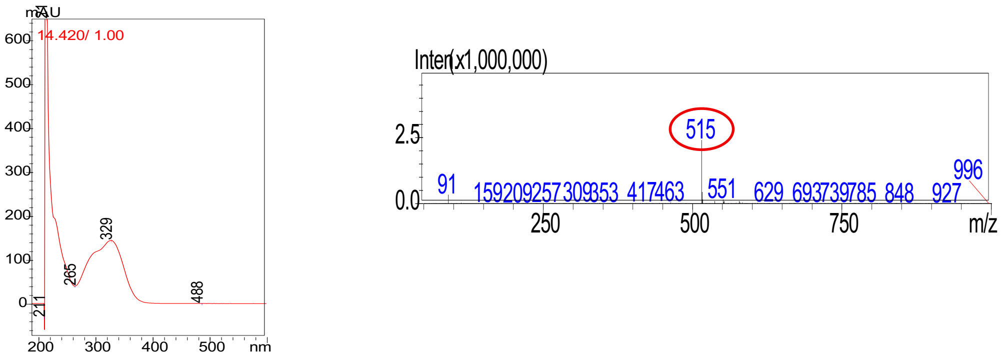

Figure S2-10. UV spectra and mass spectra of compound **9** (Rt=14.40 min) from *B. conferta*.
