# Supplementary material for: Hydrogen Peroxide-Oxidative Signaling Enhances Biosynthesis of Specialized Metabolites in Baccharis conferta Kunth
Source: Int J Mol Sci. 2026 Mar 10;27(6):2544. doi: 10.3390/ijms27062544 (PMC13027281; doi:10.3390/ijms27062544)
Supplement: Supplementary file 1 [file ijms-27-02544-s001.zip › Supplementary Data S3. Comparative LCΓÇôMS Metabolic Profiling of Baccharis conferta plants in response to Hydrogen Peroxide.pdf]

Supplementary data S3. Comparative LC–MS Metabolic Profiling of *Baccharis conferta* plants in response to Hydrogen Peroxide (H_2O_2)

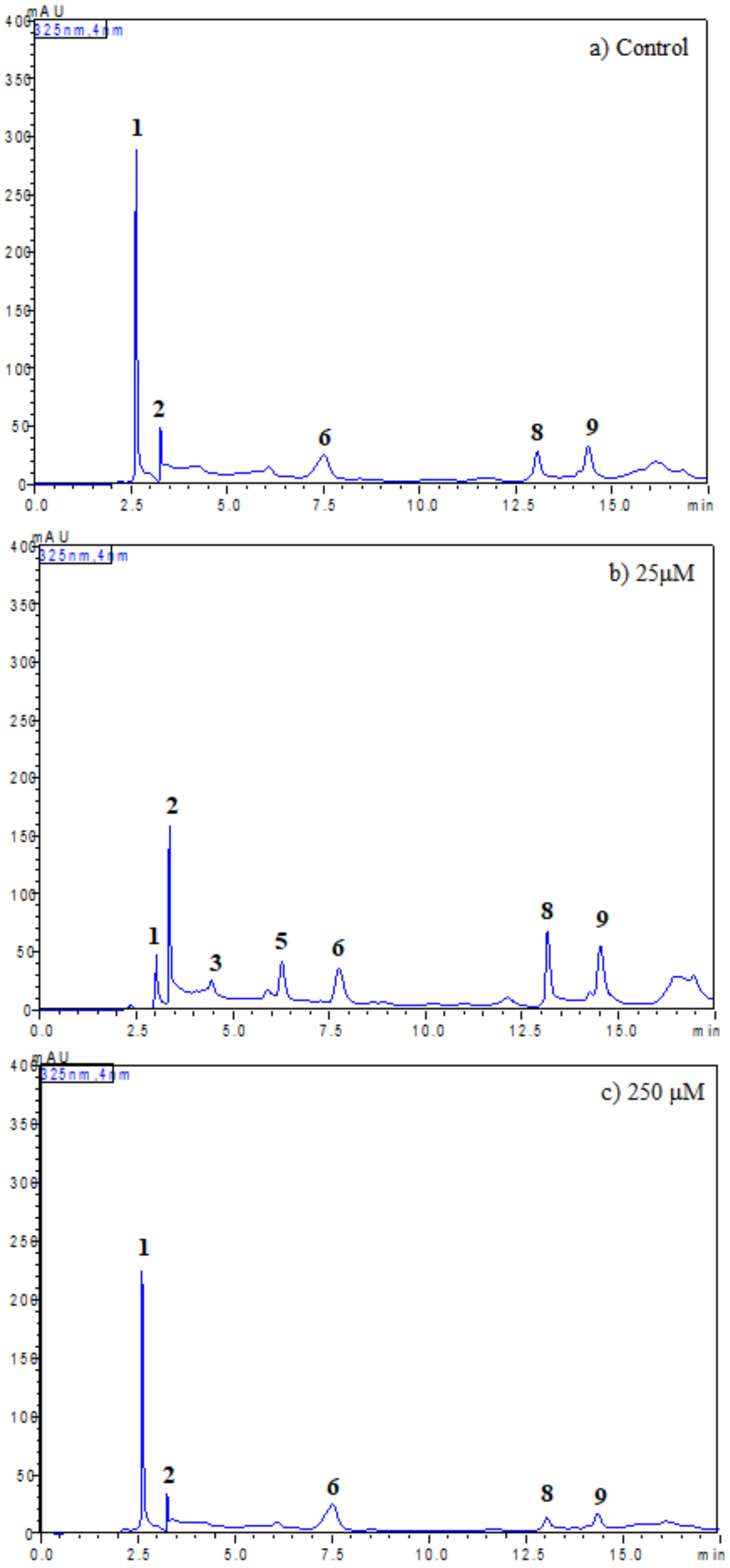

Figure S3-1. LC-MS profile of *B. conferta* plants after elicitation (9 h) with hydrogen peroxide (H_2O_2) at 325 mn. **a)** Control, **b)** 25 µM and **c)** 250 µM.

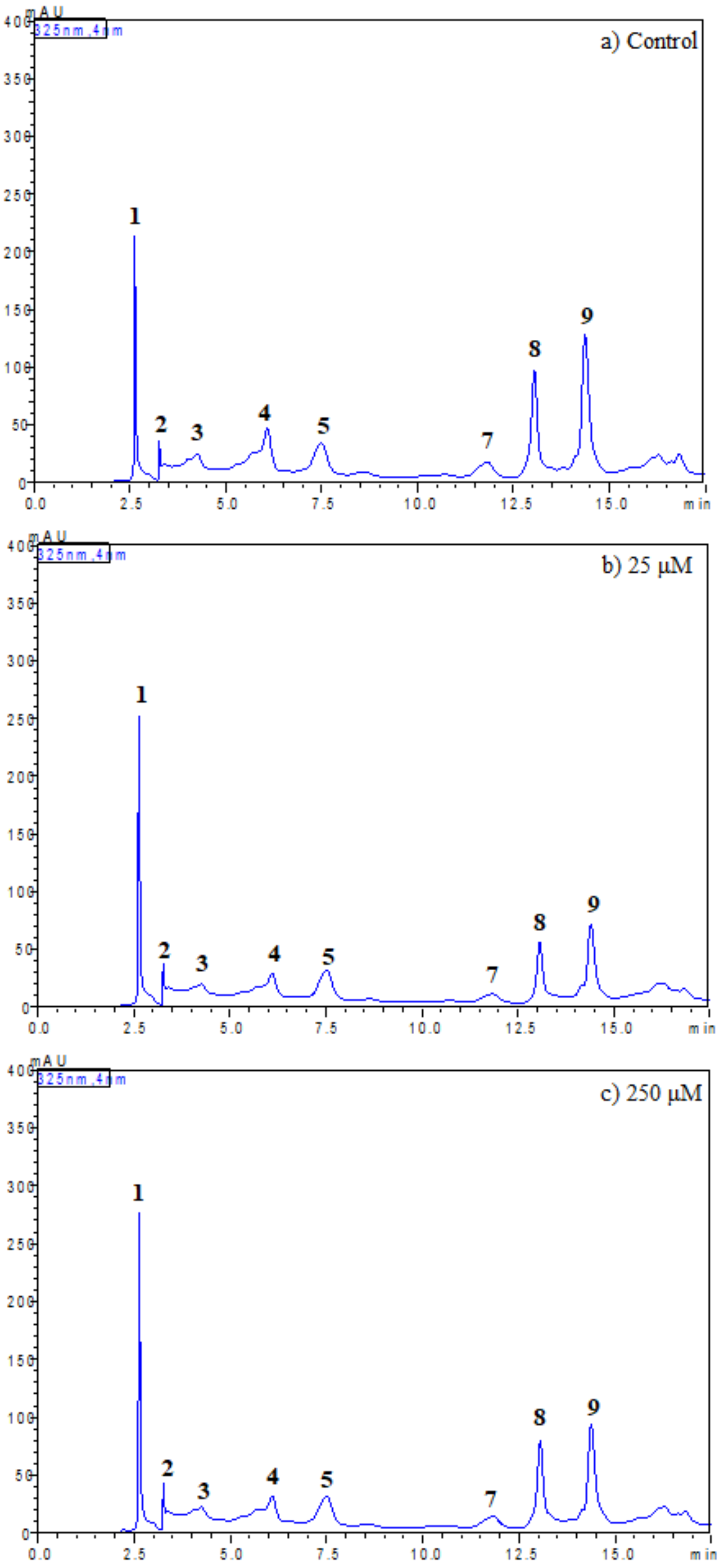

Figure S3-2. LC-MS profile of *B. conferta* plants after elicitation (24 h) with hydrogen peroxide (H_2O_2) at 325 mn. **a)** Control, **b)** 25 µM and **c)** 250 µM.

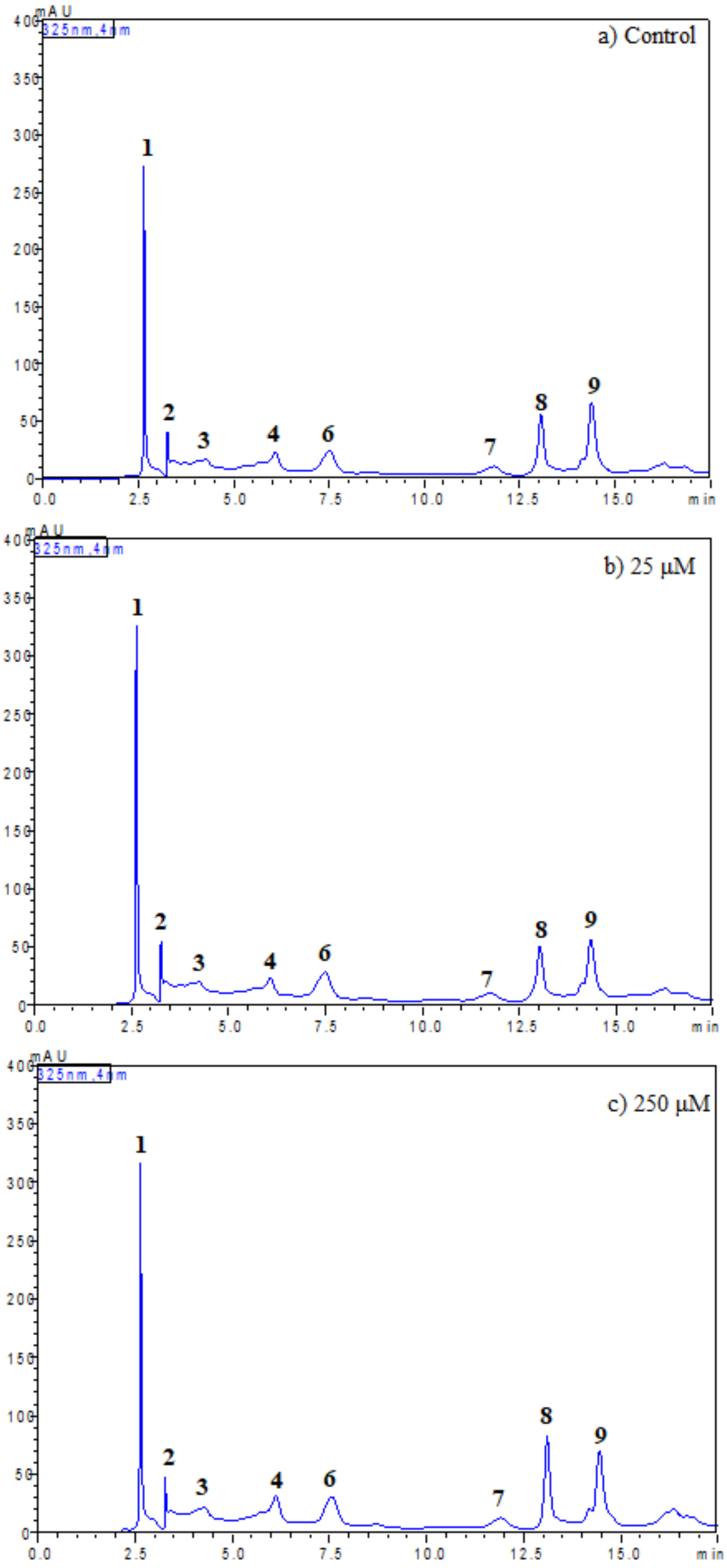

Figure S3-3. LC-MS profile of *B. conferta* plants after elicitation (48 h) with hydrogen peroxide (H_2O_2) at 325 mn. **a)** Control, **b)** 25 µM and **c)** 250 µM.
