# Supplementary material for: Hydrogen Peroxide-Oxidative Signaling Enhances Biosynthesis of Specialized Metabolites in Baccharis conferta Kunth
Source: Int J Mol Sci. 2026 Mar 10;27(6):2544. doi: 10.3390/ijms27062544 (PMC13027281; doi:10.3390/ijms27062544)
Supplement: Supplementary file 1 [file ijms-27-02544-s001.zip › Supplementary Data S5. A partial sequence of the 1-deoxy-D-xylulose-5-phosphate synthase (DXS) gene specific to B. conferta.pdf]

Supplementary Data S5. A partial sequence of the 1-deoxy-*D*-xylulose-5-phosphate synthase (DXS) gene specific to *B. conferta*

>OP047919.1 Baccharis conferta clone 2 1-deoxy-D-xylulose-5 phosphate synthase (DXS) mRNA, partial cds

GGA GAT GGT GCC ATG ACA GCT GGG CAA ACT TAC GAG GCC ATG AGA
AAG GCA GGA TTT CTT GGT GCA AAT TTA ATC ATT GTT TTA AAT GAT
AAC AAG CAA GTT ACT TTA CCA ACT GCT ACA TTA GAT GGC CCT GCA
ACT CCT GTT GGA GCT CTC AGC AGT ACT TTA GTT AAA CTT CAA GCA
AGC CCC GAA TTC CGT AAA CTT CGA GAA GCT GCC AAG AGT ATT ACA
AAG CAA ATA GGA ATT CAA ACA CAT CAA GTT GCT GCA AAA GTA GAT
GAG TAT GCA AGG GGT ATG ATT AGT GAT AAT GGG TCA ACT TTC TTT
GAG GAG CTT GGC CTC TAT TGG AAA GGT CCT GTA GAT GGG CTA ACC
TTG ATG ATC TAG TCA CAA TCT TTG AGA AAG TGA AGT CAA TGC CAG
CCC CAG GTC CAG TTC TGA TTC ACA TTG TAA CGG AGA AAG GAA AGG
GAC ACC CTC CAG CTG AAG TAG CTG CTG ACA AAA TGC ATG GAG TTG
TCA AGT TTG ATG CCG AAA CTG GAC CGC AGT TCA AGC CAA AAT CCC
CGA CAC TTT CAT ATA CAC AGT ACT TTG CTG AGG CAC TCA TAA AAG
AAG CA

X24893.1 1-deoxy-D-xylulose-5-phosphate synthase, partial [Baccharis conferta]

GDGAMTAGQTYEAMRKAGFLGANLIIVLNDNKQVTLPTATLDGPATPVGALSSTLVKLQAS
PEFRKLREAAKSITKQIGIQTHQVAAKVDEYARGMISDNGSTFFEELGLYWKGPVDGHNLD
DLVTIFEKVKSMPAPGPVLIHIVTEKGKGHPPAEVAADKMHGVVKFDAETGPQFKPKSPTL
SYTQYFAEALIKEA
