# Supplementary material for: Hydrogen Peroxide-Oxidative Signaling Enhances Biosynthesis of Specialized Metabolites in Baccharis conferta Kunth
Source: Int J Mol Sci. 2026 Mar 10;27(6):2544. doi: 10.3390/ijms27062544 (PMC13027281; doi:10.3390/ijms27062544)
Supplement: Supplementary file 1 [file ijms-27-02544-s001.zip › Supplementary Data S7. Analysis of DXS, sequence Identity Matrix.pdf]

Sequence Identity Matrix

Input Alignment File: C:\Users\lizru\Desktop\DXS-T\Alineamiento-R DXS-I-II-III.bio

Family	Sequence	Seq->	NCBI	*Baccharis conferta*	*Actinidia chinensis*	*Andrographis alata*	*Andrographis paniculata*
		Baccharis conferta	WEX24893.1	ID	71%	67%	67%
	1	*Actinidia chinensis*	AID55339.1	71%	ID	71%	71%
	2	*Andrographis alata*	UYR28655.1	67%	71%	ID	99%
	3	*Andrographis paniculata*	AUG90532.1	67%	71%	99%	ID
	4	*Aquilaria sinensis*	AHI62962.1	69%	73%	92%	92%
	5	*Arabidopsis thaliana*	AEE76516.1	69%	71%	88%	88%
	6	*Artemisia annua*	AAD56390.2	68%	71%	95%	94%
	7	*Ayapana triplinervis*	WNX96120.1	69%	72%	95%	94%
	8	*Bixa orellana*	QTZ19496.1	68%	71%	94%	94%
	9	*Camellia sinensis*	AYQ58349.1	70%	87%	70%	70%
	10	*Eucommia ulmoides*	ASK59030.1	67%	69%	92%	92%
	11	*Gardenia jasminoides*	ARU08102.1	82%	77%	73%	73%
	12	*Glycine max*	ACO72582.1	68%	72%	92%	92%
	13	*Hevea brasiliensis*	BAF98289.1	68%	71%	94%	93%
	14	*Houttuynia cordata*	AHB79076.1	73%	81%	71%	71%
	15	*Lavandula angustifolia*	AGQ04154.1	68%	69%	95%	95%
	16	*Lycium ruthenicum*	AIX87515.1	67%	70%	91%	91%
	17	*Magnolia champaca*	ART66975.1	84%	78%	71%	71%
**I**	18	*Medicago truncatula*	AES91764.1	68%	71%	91%	90%
	19	*Osmanthus fragrans*	AOT86855.1	68%	70%	97%	96%
	20	*Pinus taeda*	ACJ67021.1	69%	69%	86%	86%
	21	*Plectranthus barbatus*	ALE19960.1	67%	69%	96%	95%
	22	*Poa pratensis*	AXS78096.1	69%	74%	87%	88%
	23	*Prunella vulgaris*	QEV81817.1	71%	85%	69%	69%
	24	*Salvia fruticosa*	QCQ28843.1	69%	69%	95%	95%
	25	*Salvia divinorum*	KAL1569699.	69%	70%	95%	94%

	26	*Salvia pomifera*	QCQ28844.1	69%	69%	95%	95%
	27	*Scutellaria barbata*	QEY10166.1	67%	69%	96%	96%
	28	*Solanum tuberosum*	NP_00127513	68%	71%	92%	92%
	29	*Stevia rebaudiana*	ALJ30086.1	69%	72%	94%	93%
	30	*Taraxacum kok-saghyz*	AMB19703.1	86%	78%	73%	73%
	31	*Thuja plicata*	QGZ06734.1	68%	70%	87%	87%
	32	*Trifolium repens*	WJX93507.1	68%	71%	91%	91%
	33	*Zea mays*	ACG27905.1	70%	71%	89%	90%
	34	*Withania somnifera*	AOX15279.1	68%	71%	92%	92%
	1	*Andrographis alata*	UYR28657.1	72%	82%	69%	69%
	2	*Arabidopsis thaliana*		56%	56%	68%	68%
	3	*Ayapana triplinervis*	WNX96118.1	72%	79%	64%	64%
	4	*Camellia sinensis*	QFS18946.1	83%	78%	73%	73%
	5	*Catharanthus roseus*	ABI35993.1	69%	80%	71%	71%
	6	*Centranthera grandiflora*	AZG04488.1	10%	7%	10%	10%
	7	*Lycium barbarum*	AIX87492.1	80%	78%	74%	74%
	8	*Lycium ruthenicum*	AIX87516.1	80%	78%	75%	75%
	9	*Magnolia champaca*	ART66976.1	74%	83%	75%	75%
	10	*Medicago truncatula*	CAD22531.1	77%	74%	69%	69%
	11	*Mitragyna speciosa*	AEZ55866.1	72%	81%	73%	72%
	12	*Hevea brasiliensis*	NP_00140895	7%	7%	8%	8%
**II**	13	*Osmanthus fragrans*	AOT86856.1	7%	7%	7%	7%
	14	*Pinus massoniana*	UIB01903.1	7%	7%	8%	8%
	15	*Pinus taeda*	ACJ67020.1	6%	6%	6%	6%
	16	*Plectranthus barbatus*	AOZ60045.1	7%	6%	7%	7%
	17	*Taraxacum koksaghyz*	AMB19704.1	5%	6%	6%	6%
	18	*Thuja plicata*	QGZ06735.1	6%	6%	5%	5%
	19	*Tripterygium wilfordii*	AKP20998.1	7%	7%	8%	8%
	20	*Salvia fruticosa*	QCQ28848.1	7%	6%	7%	7%
	21	*Salvia officinalis*	QCQ28847.1	7%	6%	7%	7%
	22	*Salvia pomifera*	QCQ28849.1	7%	6%	7%	7%
	23	*Solanum lycopersicum*	NP_00133279	8%	7%	8%	8%
	24	*Stevia rebaudiana*	ALJ30087.1	7%	6%	7%	7%
	25	*Withania somnifera*	AOX15280.1	6%	7%	7%	7%
	1	*Aquilaria sinensis*	AFU75320.1	5%	6%	5%	5%

	2	*Arabidopsis thaliana*	AED91670.1	6%	7%	7%	7%
	3	*Ayapana triplinervis*	WNX96119.1	5%	6%	6%	6%
	4	*Bixa orellana*	AMJ39462.1	5%	6%	6%	6%
	5	*Magnolia champaca*	ART66977.1	6%	6%	6%	6%
	6	*Pinus massoniana*	UIB01904.1	8%	9%	9%	9%
**III**	7	*Salvia pomifera*	QCQ28854.1	7%	7%	8%	8%
	8	*Salvia fruticosa*	QCQ28853.1	7%	7%	8%	8%
	9	*Salvia officinalis*	QCQ28852.1	7%	7%	8%	8%
	10	*Stevia rebaudiana*	ALJ30088.1	5%	6%	6%	6%
	11	*Taraxacum koksaghyz*	AMB19705.1	6%	7%	7%	7%
	12	*Tripterygium wilfordii*	AKP20999.1	5%	6%	5%	5%
		Clustal Consensus		0%	0%	0%	0%

Aquilaria sinensis	***Arabidopsis thaliana***	***Artemisia annua***	***Ayapana triplinervis***	***Bixa orellana***	***Camellia sinensis***	***Eucommia ulmoides***	***Gardenia jasminoides***	***Glycine max***	***Hevea brasiliensis***
69%	69%	68%	69%	68%	70%	67%	82%	68%	68%
73%	71%	71%	72%	71%	87%	69%	77%	72%	71%
92%	88%	95%	95%	94%	70%	92%	73%	92%	94%
92%	88%	94%	94%	94%	70%	92%	73%	92%	93%
ID	90%	94%	93%	94%	74%	92%	74%	93%	94%
90%	ID	89%	89%	90%	71%	90%	74%	90%	90%
94%	89%	ID	98%	95%	70%	93%	74%	95%	95%
93%	89%	98%	ID	94%	71%	92%	74%	94%	94%
94%	90%	95%	94%	ID	71%	92%	73%	94%	97%
74%	71%	70%	71%	71%	ID	69%	77%	72%	70%
92%	90%	93%	92%	92%	69%	ID	71%	90%	92%
74%	74%	74%	74%	73%	77%	71%	ID	74%	73%
93%	90%	95%	94%	94%	72%	90%	74%	ID	95%
94%	90%	95%	94%	97%	70%	92%	73%	95%	ID
72%	72%	71%	72%	72%	81%	70%	79%	72%	71%
93%	88%	95%	93%	94%	70%	92%	73%	93%	94%
92%	89%	93%	93%	94%	70%	91%	72%	91%	93%
74%	72%	72%	73%	73%	77%	71%	85%	73%	73%
92%	90%	93%	93%	92%	70%	90%	73%	94%	94%
94%	89%	97%	96%	96%	70%	94%	73%	94%	96%
86%	83%	87%	85%	86%	68%	85%	74%	86%	87%
93%	89%	96%	95%	95%	69%	93%	73%	93%	95%
89%	87%	88%	89%	89%	72%	85%	75%	89%	90%
71%	70%	71%	72%	70%	83%	70%	79%	71%	70%
93%	88%	95%	93%	94%	70%	92%	74%	93%	94%
94%	89%	95%	94%	95%	70%	91%	75%	94%	95%

93%	88%	95%	93%	94%	70%	92%	74%	93%	94%
93%	89%	95%	93%	94%	69%	92%	73%	93%	94%
93%	90%	93%	93%	95%	71%	91%	73%	92%	94%
94%	89%	98%	99%	95%	71%	92%	74%	95%	95%
75%	74%	74%	75%	74%	77%	72%	89%	74%	74%
88%	85%	88%	86%	89%	70%	85%	73%	88%	90%
92%	90%	94%	93%	94%	71%	90%	73%	96%	95%
89%	87%	89%	89%	90%	71%	86%	75%	90%	90%
93%	91%	93%	93%	94%	71%	90%	73%	92%	95%
71%	70%	71%	71%	69%	82%	69%	79%	70%	69%
69%	69%	69%	68%	69%	57%	68%	59%	68%	68%
69%	67%	66%	66%	66%	80%	64%	76%	67%	65%
74%	73%	74%	74%	74%	78%	72%	90%	74%	74%
74%	71%	72%	73%	72%	80%	70%	76%	74%	71%
10%	9%	10%	10%	10%	7%	9%	11%	10%	10%
74%	72%	74%	75%	76%	77%	73%	91%	74%	75%
75%	73%	75%	76%	77%	77%	73%	92%	75%	76%
75%	72%	73%	74%	74%	79%	73%	78%	74%	74%
70%	69%	69%	69%	70%	71%	69%	81%	70%	69%
75%	72%	73%	74%	72%	82%	72%	79%	73%	72%
8%	8%	7%	7%	8%	6%	9%	8%	8%	8%
8%	7%	6%	6%	7%	7%	8%	8%	7%	7%
7%	7%	7%	7%	7%	7%	8%	8%	7%	7%
6%	6%	5%	5%	6%	5%	7%	6%	6%	6%
8%	7%	7%	7%	7%	6%	8%	8%	7%	7%
7%	6%	6%	6%	6%	6%	6%	6%	6%	6%
5%	6%	5%	5%	6%	6%	6%	6%	6%	5%
8%	8%	7%	7%	8%	6%	9%	8%	8%	8%
8%	7%	7%	7%	7%	6%	8%	8%	7%	7%
8%	7%	7%	7%	7%	6%	8%	8%	7%	7%
8%	7%	7%	7%	7%	6%	8%	8%	7%	7%
8%	8%	7%	7%	7%	6%	8%	9%	8%	7%
8%	7%	7%	7%	7%	6%	8%	8%	7%	7%
7%	7%	6%	6%	7%	6%	7%	6%	7%	7%
5%	6%	5%	5%	5%	6%	7%	6%	5%	5%

7%	7%	6%	6%	7%	6%	7%	7%	7%	7%
6%	6%	5%	5%	6%	6%	6%	7%	6%	6%
6%	6%	5%	5%	6%	6%	6%	6%	6%	6%
6%	6%	5%	5%	6%	6%	7%	7%	6%	6%
9%	8%	8%	8%	8%	7%	9%	9%	9%	8%
8%	8%	7%	7%	8%	6%	8%	8%	8%	7%
8%	8%	7%	7%	8%	6%	8%	8%	8%	7%
8%	8%	7%	7%	8%	6%	8%	8%	8%	7%
6%	6%	6%	6%	6%	6%	6%	7%	6%	6%
6%	6%	6%	6%	6%	6%	7%	7%	6%	6%
5%	6%	5%	5%	5%	5%	6%	6%	5%	5%
0%	0%	0%	0%	0%	0%	0%	0%	0%	0%

Family I (DXS1)

Houttuynia cordata	*Lavandula angustifolia*	*Lycium ruthenicum*	*Magnolia champaca*	*Medicago truncatula*	*Osmanthus fragrans*	*Pinus taeda*	*Plectranthus barbatus*	*Poa pratensis*	*Prunella vulgaris*
73%	68%	67%	84%	68%	68%	69%	67%	69%	71%
81%	69%	70%	78%	71%	70%	69%	69%	74%	85%
71%	95%	91%	71%	91%	97%	86%	96%	87%	69%
71%	95%	91%	71%	90%	96%	86%	95%	88%	69%
72%	93%	92%	74%	92%	94%	86%	93%	89%	71%
72%	88%	89%	72%	90%	89%	83%	89%	87%	70%
71%	95%	93%	72%	93%	97%	87%	96%	88%	71%
72%	93%	93%	73%	93%	96%	85%	95%	89%	72%
72%	94%	94%	73%	92%	96%	86%	95%	89%	70%
81%	70%	70%	77%	70%	70%	68%	69%	72%	83%
70%	92%	91%	71%	90%	94%	85%	93%	85%	70%
79%	73%	72%	85%	73%	73%	74%	73%	75%	79%
72%	93%	91%	73%	94%	94%	86%	93%	89%	71%
71%	94%	93%	73%	94%	96%	87%	95%	90%	70%
ID	69%	72%	82%	71%	71%	68%	71%	73%	82%
69%	ID	92%	71%	90%	96%	87%	97%	88%	68%
72%	92%	ID	71%	90%	94%	85%	93%	88%	69%
82%	71%	71%	ID	72%	72%	72%	71%	74%	77%
71%	90%	90%	72%	ID	92%	85%	91%	88%	70%
71%	96%	94%	72%	92%	ID	87%	98%	88%	70%
68%	87%	85%	72%	85%	87%	ID	86%	86%	70%
71%	97%	93%	71%	91%	98%	86%	ID	87%	69%
73%	88%	88%	74%	88%	88%	86%	87%	ID	73%
82%	68%	69%	77%	70%	70%	70%	69%	73%	ID
69%	98%	92%	72%	90%	96%	87%	97%	88%	68%
70%	98%	93%	72%	91%	96%	87%	96%	88%	68%

69%	98%	92%	72%	90%	96%	87%	97%	88%	68%
70%	96%	91%	71%	90%	96%	86%	97%	88%	68%
72%	93%	98%	72%	92%	94%	86%	93%	89%	69%
72%	94%	93%	73%	93%	96%	86%	96%	89%	72%
82%	74%	73%	90%	73%	74%	75%	73%	76%	79%
70%	90%	88%	72%	86%	89%	90%	88%	87%	69%
71%	92%	90%	73%	97%	93%	86%	92%	88%	70%
73%	89%	90%	73%	88%	90%	85%	89%	94%	71%
72%	92%	96%	72%	92%	94%	87%	93%	90%	69%
82%	68%	69%	77%	70%	70%	69%	69%	70%	85%
56%	69%	68%	57%	68%	68%	65%	68%	66%	56%
78%	64%	65%	75%	65%	65%	64%	64%	68%	78%
81%	73%	72%	87%	74%	73%	73%	73%	75%	81%
76%	71%	70%	75%	72%	71%	71%	71%	75%	78%
8%	9%	10%	10%	10%	10%	10%	10%	11%	9%
81%	74%	76%	84%	73%	75%	74%	74%	76%	79%
81%	75%	76%	85%	74%	76%	74%	75%	76%	80%
84%	73%	72%	81%	73%	73%	73%	73%	74%	81%
76%	69%	68%	83%	68%	69%	69%	68%	71%	76%
80%	72%	72%	77%	72%	73%	72%	72%	75%	84%
7%	8%	8%	8%	8%	8%	8%	8%	7%	7%
7%	7%	7%	8%	7%	7%	7%	7%	6%	7%
8%	7%	7%	7%	7%	7%	7%	7%	8%	7%
6%	6%	6%	6%	6%	6%	6%	6%	7%	7%
8%	7%	7%	8%	8%	7%	7%	7%	7%	7%
6%	6%	6%	6%	6%	6%	5%	6%	7%	6%
7%	5%	5%	7%	5%	5%	5%	5%	6%	7%
7%	8%	8%	9%	8%	8%	8%	8%	7%	7%
8%	7%	7%	8%	7%	7%	7%	7%	7%	7%
8%	7%	7%	8%	7%	7%	7%	7%	7%	7%
8%	7%	7%	8%	7%	7%	7%	7%	7%	7%
8%	7%	7%	9%	8%	7%	7%	7%	7%	7%
8%	7%	7%	8%	7%	7%	8%	7%	7%	7%
7%	7%	7%	7%	7%	7%	6%	7%	7%	8%
6%	5%	5%	6%	6%	5%	5%	5%	6%	7%

7%	7%	7%	6%	6%	7%	6%	7%	8%	8%
6%	6%	6%	6%	6%	6%	5%	6%	6%	7%
6%	6%	6%	6%	6%	6%	5%	6%	6%	6%
7%	6%	6%	7%	6%	6%	5%	6%	6%	7%
9%	8%	8%	9%	8%	8%	9%	8%	9%	8%
7%	7%	7%	8%	8%	7%	7%	7%	7%	7%
7%	7%	7%	8%	8%	7%	7%	7%	7%	7%
7%	7%	7%	8%	8%	7%	7%	7%	7%	7%
6%	6%	6%	6%	6%	6%	5%	6%	7%	7%
7%	6%	6%	6%	6%	6%	6%	6%	7%	7%
5%	5%	5%	5%	6%	5%	5%	5%	6%	7%
0%	0%	0%	0%	0%	0%	0%	0%	0%	0%

Salvia fruticosa	*Salvia divinorum*	*Salvia pomifera*	*Scutellaria barbata*	*Solanum tuberosum*	*Stevia rebaudiana*	*Taraxacum koksaghyz*	*Thuja plicata*	*Trifolium repens*	*Zea mays*
69%	69%	69%	67%	68%	69%	86%	68%	68%	70%
69%	70%	69%	69%	71%	72%	78%	70%	71%	71%
95%	95%	95%	96%	92%	94%	73%	87%	91%	89%
95%	94%	95%	96%	92%	93%	73%	87%	91%	90%
93%	94%	93%	93%	93%	94%	75%	88%	92%	89%
88%	89%	88%	89%	90%	89%	74%	85%	90%	87%
95%	95%	95%	95%	93%	98%	74%	88%	94%	89%
93%	94%	93%	93%	93%	99%	75%	86%	93%	89%
94%	95%	94%	94%	95%	95%	74%	89%	94%	90%
70%	70%	70%	69%	71%	71%	77%	70%	71%	71%
92%	91%	92%	92%	91%	92%	72%	85%	90%	86%
74%	75%	74%	73%	73%	74%	89%	73%	73%	75%
93%	94%	93%	93%	92%	95%	74%	88%	96%	90%
94%	95%	94%	94%	94%	95%	74%	90%	95%	90%
69%	70%	69%	70%	72%	72%	82%	70%	71%	73%
98%	98%	98%	96%	93%	94%	74%	90%	92%	89%
92%	93%	92%	91%	98%	93%	73%	88%	90%	90%
72%	72%	72%	71%	72%	73%	90%	72%	73%	73%
90%	91%	90%	90%	92%	93%	73%	86%	97%	88%
96%	96%	96%	96%	94%	96%	74%	89%	93%	90%
87%	87%	87%	86%	86%	86%	75%	90%	86%	85%
97%	96%	97%	97%	93%	96%	73%	88%	92%	89%
88%	88%	88%	88%	89%	89%	76%	87%	88%	94%
68%	68%	68%	68%	69%	72%	79%	69%	70%	71%
ID	99%	100%	96%	93%	94%	74%	90%	92%	89%
99%	ID	99%	96%	94%	95%	75%	90%	92%	89%

100%	99% ID		96%	93%	94%	74%	90%	92%	89%
96%	96%	96% ID		92%	94%	73%	89%	92%	88%
93%	94%	93%	92% ID		93%	74%	89%	92%	91%
94%	95%	94%	94%	93% ID		75%	87%	94%	89%
74%	75%	74%	73%	74%	75% ID		74%	74%	75%
90%	90%	90%	89%	89%	87%	74% ID		87%	87%
92%	92%	92%	92%	92%	94%	74%	87% ID		87%
89%	89%	89%	88%	91%	89%	75%	87%	87% ID	
92%	93%	92%	93%	97%	93%	74%	90%	92%	92%
68%	68%	68%	69%	69%	71%	79%	69%	69%	70%
69%	69%	69%	68%	69%	68%	60%	67%	68%	68%
65%	66%	65%	65%	66%	66%	77%	65%	65%	68%
73%	74%	73%	73%	73%	74%	90%	74%	74%	76%
71%	72%	71%	71%	71%	73%	76%	72%	72%	74%
10%	10%	10%	10%	10%	10%	10%	10%	10%	11%
74%	75%	74%	74%	76%	75%	89%	74%	74%	76%
75%	76%	75%	75%	77%	76%	89%	75%	75%	77%
73%	74%	73%	74%	73%	74%	81%	74%	74%	73%
69%	69%	69%	69%	69%	69%	83%	70%	69%	71%
73%	73%	73%	74%	73%	74%	79%	72%	72%	73%
8%	8%	8%	8%	8%	7%	6%	8%	8%	8%
7%	7%	7%	7%	7%	6%	7%	7%	7%	7%
8%	8%	8%	7%	7%	7%	7%	7%	8%	8%
6%	6%	6%	6%	6%	5%	6%	5%	6%	7%
8%	8%	8%	7%	7%	7%	7%	7%	8%	8%
6%	6%	6%	6%	6%	6%	5%	5%	6%	7%
5%	5%	5%	5%	5%	5%	6%	5%	5%	6%
8%	8%	8%	8%	8%	7%	7%	8%	8%	8%
8%	8%	8%	7%	7%	7%	6%	7%	7%	8%
8%	8%	8%	7%	7%	7%	6%	7%	7%	8%
8%	8%	8%	7%	7%	7%	6%	7%	7%	8%
8%	8%	8%	7%	7%	7%	7%	7%	7%	8%
8%	8%	8%	7%	7%	7%	7%	8%	7%	8%
7%	7%	7%	7%	7%	6%	6%	6%	6%	7%
5%	5%	5%	5%	5%	5%	5%	5%	5%	6%

7%	7%	7%	7%	7%	6%	6%	6%	6%	8%
6%	6%	6%	6%	6%	5%	5%	5%	6%	7%
6%	6%	6%	6%	6%	5%	5%	5%	5%	6%
6%	6%	6%	6%	6%	5%	5%	5%	6%	6%
9%	9%	9%	8%	8%	8%	8%	8%	8%	9%
8%	8%	8%	7%	7%	7%	7%	7%	7%	7%
8%	8%	8%	7%	7%	7%	7%	7%	7%	7%
8%	8%	8%	7%	7%	7%	7%	7%	7%	7%
7%	7%	7%	6%	6%	6%	5%	5%	6%	7%
7%	7%	7%	6%	6%	6%	6%	6%	7%	7%
6%	6%	6%	5%	5%	5%	5%	5%	6%	6%
0%	0%	0%	0%	0%	0%	0%	0%	0%	0%

Withania somnifera	***Andrographis alata***	***Arabidopsis thaliana***	***Ayapana triplinervis***	***Camellia sinensis***	***Catharanthus roseus***	***Centranthera grandiflora***	***Lycium barbarum***	***Lycium ruthenicum***
68%	72%	56%	72%	83%	69%	10%	80%	80%
71%	82%	56%	79%	78%	80%	7%	78%	78%
92%	69%	68%	64%	73%	71%	10%	74%	75%
92%	69%	68%	64%	73%	71%	10%	74%	75%
93%	71%	69%	69%	74%	74%	10%	74%	75%
91%	70%	69%	67%	73%	71%	9%	72%	73%
93%	71%	69%	66%	74%	72%	10%	74%	75%
93%	71%	68%	66%	74%	73%	10%	75%	76%
94%	69%	69%	66%	74%	72%	10%	76%	77%
71%	82%	57%	80%	78%	80%	7%	77%	77%
90%	69%	68%	64%	72%	70%	9%	73%	73%
73%	79%	59%	76%	90%	76%	11%	91%	92%
92%	70%	68%	67%	74%	74%	10%	74%	75%
95%	69%	68%	65%	74%	71%	10%	75%	76%
72%	82%	56%	78%	81%	76%	8%	81%	81%
92%	68%	69%	64%	73%	71%	9%	74%	75%
96%	69%	68%	65%	72%	70%	10%	76%	76%
72%	77%	57%	75%	87%	75%	10%	84%	85%
92%	70%	68%	65%	74%	72%	10%	73%	74%
94%	70%	68%	65%	73%	71%	10%	75%	76%
87%	69%	65%	64%	73%	71%	10%	74%	74%
93%	69%	68%	64%	73%	71%	10%	74%	75%
90%	70%	66%	68%	75%	75%	11%	76%	76%
69%	85%	56%	78%	81%	78%	9%	79%	80%
92%	68%	69%	65%	73%	71%	10%	74%	75%
93%	68%	69%	66%	74%	72%	10%	75%	76%

92%	68%	69%	65%	73%	71%	10%	74%	75%
93%	69%	68%	65%	73%	71%	10%	74%	75%
97%	69%	69%	66%	73%	71%	10%	76%	77%
93%	71%	68%	66%	74%	73%	10%	75%	76%
74%	79%	60%	77%	90%	76%	10%	89%	89%
90%	69%	67%	65%	74%	72%	10%	74%	75%
92%	69%	68%	65%	74%	72%	10%	74%	75%
92%	70%	68%	68%	76%	74%	11%	76%	77%
ID	70%	70%	67%	73%	72%	10%	76%	77%
70%	ID	56%	79%	79%	80%	8%	78%	79%
70%	56%	ID	55%	60%	58%	9%	59%	59%
67%	79%	55%	ID	75%	75%	9%	73%	74%
73%	79%	60%	75%	ID	76%	10%	88%	89%
72%	80%	58%	75%	76%	ID	7%	75%	76%
10%	8%	9%	9%	10%	7%	ID	10%	10%
76%	78%	59%	73%	88%	75%	10%	ID	98%
77%	79%	59%	74%	89%	76%	10%	98%	ID
73%	78%	57%	78%	80%	77%	9%	79%	79%
69%	72%	55%	72%	83%	73%	10%	82%	83%
73%	81%	57%	78%	78%	80%	6%	78%	79%
8%	6%	6%	6%	8%	7%	4%	7%	7%
7%	6%	5%	7%	8%	7%	4%	7%	8%
7%	7%	5%	6%	8%	7%	5%	8%	7%
6%	6%	4%	5%	7%	5%	5%	6%	6%
7%	6%	6%	6%	8%	7%	5%	7%	8%
6%	5%	6%	5%	6%	6%	4%	6%	6%
5%	5%	4%	6%	6%	6%	4%	6%	6%
8%	6%	6%	7%	8%	7%	3%	7%	8%
7%	6%	5%	6%	8%	7%	5%	7%	7%
7%	6%	5%	6%	8%	7%	5%	7%	7%
7%	6%	5%	6%	8%	7%	5%	7%	7%
7%	6%	6%	7%	9%	8%	5%	8%	8%
7%	6%	5%	6%	8%	8%	4%	7%	7%
7%	6%	6%	7%	6%	6%	5%	7%	7%
5%	5%	5%	6%	6%	6%	6%	6%	6%

7%	6%	6%	6%	6%	7%	4%	7%	7%
6%	7%	5%	5%	6%	6%	5%	7%	7%
6%	5%	6%	6%	6%	6%	5%	6%	6%
6%	5%	4%	6%	7%	6%	6%	7%	7%
8%	8%	6%	8%	9%	9%	5%	9%	9%
7%	6%	6%	7%	8%	7%	4%	7%	8%
7%	6%	6%	7%	8%	7%	4%	7%	8%
7%	6%	6%	7%	8%	7%	4%	7%	8%
6%	6%	6%	6%	6%	6%	5%	7%	7%
6%	6%	5%	6%	7%	6%	5%	6%	6%
5%	5%	5%	6%	6%	5%	6%	6%	6%
0%	0%	0%	0%	0%	0%	0%	0%	0%

Family II (DXS2)

Magnolia champaca	*Medicago truncatula*	*Mitragyna speciosa*	*Hevea brasiliensis*	*Osmanthus fragrans*	*Pinus massoniana*	*Pinus taeda*	*Plectranthus barbatus*	*Taraxacum koksaghyz*
74%	77%	72%	7%	7%	7%	6%	7%	5%
83%	74%	81%	7%	7%	7%	6%	6%	6%
75%	69%	73%	8%	7%	8%	6%	7%	6%
75%	69%	72%	8%	7%	8%	6%	7%	6%
75%	70%	75%	8%	8%	7%	6%	8%	7%
72%	69%	72%	8%	7%	7%	6%	7%	6%
73%	69%	73%	7%	6%	7%	5%	7%	6%
74%	69%	74%	7%	6%	7%	5%	7%	6%
74%	70%	72%	8%	7%	7%	6%	7%	6%
79%	71%	82%	6%	7%	7%	5%	6%	6%
73%	69%	72%	9%	8%	8%	7%	8%	6%
78%	81%	79%	8%	8%	8%	6%	8%	6%
74%	70%	73%	8%	7%	7%	6%	7%	6%
74%	69%	72%	8%	7%	7%	6%	7%	6%
84%	76%	80%	7%	7%	8%	6%	8%	6%
73%	69%	72%	8%	7%	7%	6%	7%	6%
72%	68%	72%	8%	7%	7%	6%	7%	6%
81%	83%	77%	8%	8%	7%	6%	8%	6%
73%	68%	72%	8%	7%	7%	6%	8%	6%
73%	69%	73%	8%	7%	7%	6%	7%	6%
73%	69%	72%	8%	7%	7%	6%	7%	5%
73%	68%	72%	8%	7%	7%	6%	7%	6%
74%	71%	75%	7%	6%	8%	7%	7%	7%
81%	76%	84%	7%	7%	7%	7%	7%	6%
73%	69%	73%	8%	7%	8%	6%	8%	6%
74%	69%	73%	8%	7%	8%	6%	8%	6%

73%	69%	73%	8%	7%	8%	6%	8%	6%
74%	69%	74%	8%	7%	7%	6%	7%	6%
73%	69%	73%	8%	7%	7%	6%	7%	6%
74%	69%	74%	7%	6%	7%	5%	7%	6%
81%	83%	79%	6%	7%	7%	6%	7%	5%
74%	70%	72%	8%	7%	7%	5%	7%	5%
74%	69%	72%	8%	7%	8%	6%	8%	6%
73%	71%	73%	8%	7%	8%	7%	8%	7%
73%	69%	73%	8%	7%	7%	6%	7%	6%
78%	72%	81%	6%	6%	7%	6%	6%	5%
57%	55%	57%	6%	5%	5%	4%	6%	6%
78%	72%	78%	6%	7%	6%	5%	6%	5%
80%	83%	78%	8%	8%	8%	7%	8%	6%
77%	73%	80%	7%	7%	7%	5%	7%	6%
9%	10%	6%	4%	4%	5%	5%	5%	4%
79%	82%	78%	7%	7%	8%	6%	7%	6%
79%	83%	79%	7%	8%	7%	6%	8%	6%
ID	77%	79%	7%	8%	7%	6%	7%	6%
77%	ID	75%	7%	8%	7%	6%	8%	6%
79%	75%	ID	7%	8%	6%	5%	7%	6%
7%	7%	7%	ID	89%	78%	77%	79%	56%
8%	8%	8%	89%	ID	74%	73%	79%	56%
7%	7%	6%	78%	74%	ID	84%	76%	56%
6%	6%	5%	77%	73%	84%	ID	74%	58%
7%	8%	7%	79%	79%	76%	74%	ID	51%
6%	6%	6%	56%	56%	56%	58%	51%	ID
6%	8%	6%	62%	60%	60%	62%	57%	67%
8%	8%	7%	92%	89%	77%	75%	80%	58%
7%	7%	7%	78%	78%	76%	74%	97%	50%
7%	7%	7%	78%	78%	76%	74%	97%	50%
7%	7%	7%	78%	78%	76%	74%	97%	50%
8%	8%	7%	89%	88%	79%	78%	81%	58%
7%	7%	8%	74%	75%	73%	72%	81%	52%
7%	7%	6%	56%	54%	55%	56%	51%	80%
6%	6%	6%	58%	56%	54%	57%	49%	77%

7%	8%	7%	58%	56%	56%	57%	51%	76%
5%	7%	6%	55%	53%	56%	56%	52%	87%
5%	7%	6%	59%	59%	56%	59%	53%	84%
6%	7%	6%	61%	62%	60%	61%	56%	82%
8%	8%	8%	80%	76%	78%	81%	78%	55%
8%	8%	7%	90%	92%	78%	77%	83%	57%
8%	8%	7%	90%	92%	78%	77%	83%	57%
8%	8%	7%	90%	92%	78%	77%	83%	57%
5%	7%	6%	57%	56%	58%	58%	54%	87%
6%	7%	6%	74%	73%	73%	79%	69%	60%
5%	6%	5%	59%	59%	57%	60%	54%	83%
0%	0%	0%	0%	0%	0%	0%	0%	0%

Thuja plicata	*Tripterygium wilfordii*	*Salvia fruticosa*	*Salvia officinalis*	*Salvia pomifera*	*Solanum lycopersicum*	*Stevia rebaudiana*	*Withania somnifera*	*Aquilaria sinensis*
6%	7%	7%	7%	7%	8%	7%	6%	5%
6%	7%	6%	6%	6%	7%	6%	7%	6%
5%	8%	7%	7%	7%	8%	7%	7%	5%
5%	8%	7%	7%	7%	8%	7%	7%	5%
5%	8%	8%	8%	8%	8%	8%	7%	5%
6%	8%	7%	7%	7%	8%	7%	7%	6%
5%	7%	7%	7%	7%	7%	7%	6%	5%
5%	7%	7%	7%	7%	7%	7%	6%	5%
6%	8%	7%	7%	7%	7%	7%	7%	5%
6%	6%	6%	6%	6%	6%	6%	6%	6%
6%	9%	8%	8%	8%	8%	8%	7%	7%
6%	8%	8%	8%	8%	9%	8%	6%	6%
6%	8%	7%	7%	7%	8%	7%	7%	5%
5%	8%	7%	7%	7%	7%	7%	7%	5%
7%	7%	8%	8%	8%	8%	8%	7%	6%
5%	8%	7%	7%	7%	7%	7%	7%	5%
5%	8%	7%	7%	7%	7%	7%	7%	5%
7%	9%	8%	8%	8%	9%	8%	7%	6%
5%	8%	7%	7%	7%	8%	7%	7%	6%
5%	8%	7%	7%	7%	7%	7%	7%	5%
5%	8%	7%	7%	7%	7%	8%	6%	5%
5%	8%	7%	7%	7%	7%	7%	7%	5%
6%	7%	7%	7%	7%	7%	7%	7%	6%
7%	7%	7%	7%	7%	7%	7%	8%	7%
5%	8%	8%	8%	8%	8%	8%	7%	5%
5%	8%	8%	8%	8%	8%	8%	7%	5%

	5%	8%	8%	8%	8%	8%	8%	7%	5%
	5%	8%	7%	7%	7%	7%	7%	7%	5%
	5%	8%	7%	7%	7%	7%	7%	7%	5%
	5%	7%	7%	7%	7%	7%	7%	6%	5%
	6%	7%	6%	6%	6%	7%	7%	6%	5%
	5%	8%	7%	7%	7%	7%	8%	6%	5%
	5%	8%	7%	7%	7%	7%	7%	6%	5%
	6%	8%	8%	8%	8%	8%	8%	7%	6%
	5%	8%	7%	7%	7%	7%	7%	7%	5%
	5%	6%	6%	6%	6%	6%	6%	6%	5%
	4%	6%	5%	5%	5%	6%	5%	6%	5%
	6%	7%	6%	6%	6%	7%	6%	7%	6%
	6%	8%	8%	8%	8%	9%	8%	6%	6%
	6%	7%	7%	7%	7%	8%	8%	6%	6%
	4%	3%	5%	5%	5%	5%	4%	5%	6%
	6%	7%	7%	7%	7%	8%	7%	7%	6%
	6%	8%	7%	7%	7%	8%	7%	7%	6%
	6%	8%	7%	7%	7%	8%	7%	7%	6%
	8%	8%	7%	7%	7%	8%	7%	7%	6%
	6%	7%	7%	7%	7%	7%	8%	6%	6%
	62%	92%	78%	78%	78%	89%	74%	56%	58%
	60%	89%	78%	78%	78%	88%	75%	54%	56%
	60%	77%	76%	76%	76%	79%	73%	55%	54%
	62%	75%	74%	74%	74%	78%	72%	56%	57%
	57%	80%	97%	97%	97%	81%	81%	51%	49%
	67%	58%	50%	50%	50%	58%	52%	80%	77%
ID		62%	57%	57%	57%	61%	56%	69%	67%
	62% ID		79%	79%	79%	88%	75%	57%	59%
	57%	79% ID		100%	100%	79%	80%	50%	49%
	57%	79%	100% ID		100%	79%	80%	50%	49%
	57%	79%	100%	100% ID		79%	80%	50%	49%
	61%	88%	79%	79%	79% ID		74%	57%	57%
	56%	75%	80%	80%	80%	74% ID		52%	51%
	69%	57%	50%	50%	50%	57%	52% ID		79%
	67%	59%	49%	49%	49%	57%	51%	79% ID	

67%	58%	51%	51%	51%	57%	53%	77%	74%
67%	55%	51%	51%	51%	56%	52%	78%	74%
72%	60%	53%	53%	53%	60%	54%	79%	83%
76%	63%	55%	55%	55%	62%	56%	78%	80%
62%	77%	77%	77%	77%	78%	75%	53%	53%
61%	88%	82%	82%	82%	91%	77%	57%	56%
61%	88%	82%	82%	82%	91%	77%	57%	56%
61%	88%	82%	82%	82%	91%	77%	57%	56%
67%	57%	53%	53%	53%	58%	54%	78%	72%
65%	73%	69%	69%	69%	74%	69%	58%	58%
71%	59%	54%	54%	54%	61%	55%	76%	81%
0%	0%	0%	0%	0%	0%	0%	0%	0%

Family III (DXS3)

Arabidopsis thaliana	*Ayapana triplinervis*	*Bixa orellana*	*Magnolia champaca*	*Pinus massoniana*	*Salvia pomifera*	*Salvia fruticosa*	*Salvia officinalis*	*Stevia rebaudiana*	*Taraxacum koksaghyz*
6%	5%	5%	6%	8%	7%	7%	7%	5%	6%
7%	6%	6%	6%	9%	7%	7%	7%	6%	7%
7%	6%	6%	6%	9%	8%	8%	8%	6%	7%
7%	6%	6%	6%	9%	8%	8%	8%	6%	7%
7%	6%	6%	6%	9%	8%	8%	8%	6%	6%
7%	6%	6%	6%	8%	8%	8%	8%	6%	6%
6%	5%	5%	5%	8%	7%	7%	7%	6%	6%
6%	5%	5%	5%	8%	7%	7%	7%	6%	6%
7%	6%	6%	6%	8%	8%	8%	8%	6%	6%
6%	6%	6%	6%	7%	6%	6%	6%	6%	6%
7%	6%	6%	7%	9%	8%	8%	8%	6%	7%
7%	7%	6%	7%	9%	8%	8%	8%	7%	7%
7%	6%	6%	6%	9%	8%	8%	8%	6%	6%
7%	6%	6%	6%	8%	7%	7%	7%	6%	6%
7%	6%	6%	7%	9%	7%	7%	7%	6%	7%
7%	6%	6%	6%	8%	7%	7%	7%	6%	6%
7%	6%	6%	6%	8%	7%	7%	7%	6%	6%
6%	6%	6%	7%	9%	8%	8%	8%	6%	6%
6%	6%	6%	6%	8%	8%	8%	8%	6%	6%
7%	6%	6%	6%	8%	7%	7%	7%	6%	6%
6%	5%	5%	5%	9%	7%	7%	7%	5%	6%
7%	6%	6%	6%	8%	7%	7%	7%	6%	6%
8%	6%	6%	6%	9%	7%	7%	7%	7%	7%
8%	7%	6%	7%	8%	7%	7%	7%	7%	7%
7%	6%	6%	6%	9%	8%	8%	8%	7%	7%
7%	6%	6%	6%	9%	8%	8%	8%	7%	7%

7%	6%	6%	6%	9%	8%	8%	8%	7%	7%
7%	6%	6%	6%	8%	7%	7%	7%	6%	6%
7%	6%	6%	6%	8%	7%	7%	7%	6%	6%
6%	5%	5%	5%	8%	7%	7%	7%	6%	6%
6%	5%	5%	5%	8%	7%	7%	7%	5%	6%
6%	5%	5%	5%	8%	7%	7%	7%	5%	6%
6%	6%	5%	6%	8%	7%	7%	7%	6%	7%
8%	7%	6%	6%	9%	7%	7%	7%	7%	7%
7%	6%	6%	6%	8%	7%	7%	7%	6%	6%
6%	7%	5%	5%	8%	6%	6%	6%	6%	6%
6%	5%	6%	4%	6%	6%	6%	6%	6%	5%
6%	5%	6%	6%	8%	7%	7%	7%	6%	6%
6%	6%	6%	7%	9%	8%	8%	8%	6%	7%
7%	6%	6%	6%	9%	7%	7%	7%	6%	6%
4%	5%	5%	6%	5%	4%	4%	4%	5%	5%
7%	7%	6%	7%	9%	7%	7%	7%	7%	6%
7%	7%	6%	7%	9%	8%	8%	8%	7%	6%
7%	5%	5%	6%	8%	8%	8%	8%	5%	6%
8%	7%	7%	7%	8%	8%	8%	8%	7%	7%
7%	6%	6%	6%	8%	7%	7%	7%	6%	6%
58%	55%	59%	61%	80%	90%	90%	90%	57%	74%
56%	53%	59%	62%	76%	92%	92%	92%	56%	73%
56%	56%	56%	60%	78%	78%	78%	78%	58%	73%
57%	56%	59%	61%	81%	77%	77%	77%	58%	79%
51%	52%	53%	56%	78%	83%	83%	83%	54%	69%
76%	87%	84%	82%	55%	57%	57%	57%	87%	60%
67%	67%	72%	76%	62%	61%	61%	61%	67%	65%
58%	55%	60%	63%	77%	88%	88%	88%	57%	73%
51%	51%	53%	55%	77%	82%	82%	82%	53%	69%
51%	51%	53%	55%	77%	82%	82%	82%	53%	69%
51%	51%	53%	55%	77%	82%	82%	82%	53%	69%
57%	56%	60%	62%	78%	91%	91%	91%	58%	74%
53%	52%	54%	56%	75%	77%	77%	77%	54%	69%
77%	78%	79%	78%	53%	57%	57%	57%	78%	58%
74%	74%	83%	80%	53%	56%	56%	56%	72%	58%

ID	74%	76%	76%	55%	58%	58%	58%	73%	59%
74%	ID	79%	76%	55%	55%	55%	55%	94%	59%
76%	79%	ID	86%	55%	59%	59%	59%	78%	63%
76%	76%	86%	ID	60%	62%	62%	62%	75%	65%
55%	55%	55%	60%	ID	80%	80%	80%	56%	74%
58%	55%	59%	62%	80%	ID	100%	100%	58%	75%
58%	55%	59%	62%	80%	100%	ID	100%	58%	75%
58%	55%	59%	62%	80%	100%	100%	ID	58%	75%
73%	94%	78%	75%	56%	58%	58%	58%	ID	61%
59%	59%	63%	65%	74%	75%	75%	75%	61%	ID
74%	77%	88%	87%	57%	60%	60%	60%	77%	63%
0%	0%	0%	0%	0%	0%	0%	0%	0%	0%

Tripterygium wilfordii	***Clustal Consensus***
5%	0%
6%	0%
5%	0%
5%	0%
5%	0%
6%	0%
5%	0%
5%	0%
5%	0%
5%	0%
6%	0%
6%	0%
5%	0%
5%	0%
5%	0%
5%	0%
5%	0%
5%	0%
6%	0%
5%	0%
5%	0%
5%	0%
6%	0%
7%	0%
6%	0%
6%	0%

6%	0%
5%	0%
5%	0%
5%	0%
5%	0%
5%	0%
6%	0%
6%	0%
5%	0%
5%	0%
5%	0%
6%	0%
6%	0%
5%	0%
6%	0%
6%	0%
6%	0%
5%	0%
6%	0%
5%	0%
59%	0%
59%	0%
57%	0%
60%	0%
54%	0%
83%	0%
71%	0%
59%	0%
54%	0%
54%	0%
54%	0%
61%	0%
55%	0%
76%	0%
81%	0%

	74%	0%
	77%	0%
	88%	0%
	87%	0%
	57%	0%
	60%	0%
	60%	0%
	60%	0%
	77%	0%
	63%	0%
ID		0%
	0%	ID
