# Supplementary material for: Hydrogen Peroxide-Oxidative Signaling Enhances Biosynthesis of Specialized Metabolites in Baccharis conferta Kunth
Source: Int J Mol Sci. 2026 Mar 10;27(6):2544. doi: 10.3390/ijms27062544 (PMC13027281; doi:10.3390/ijms27062544)
Supplement: Supplementary file 1 [file ijms-27-02544-s001.zip › Supplementary Data S8. HPTLC-Based confirmation of Terpene presence in Baccharis conferta dichloromethane extracts.pdf]

Supplementary Data S8. HPTLC-Based confirmation of Terpene presence in *Baccharis conferta* dichloromethane extracts

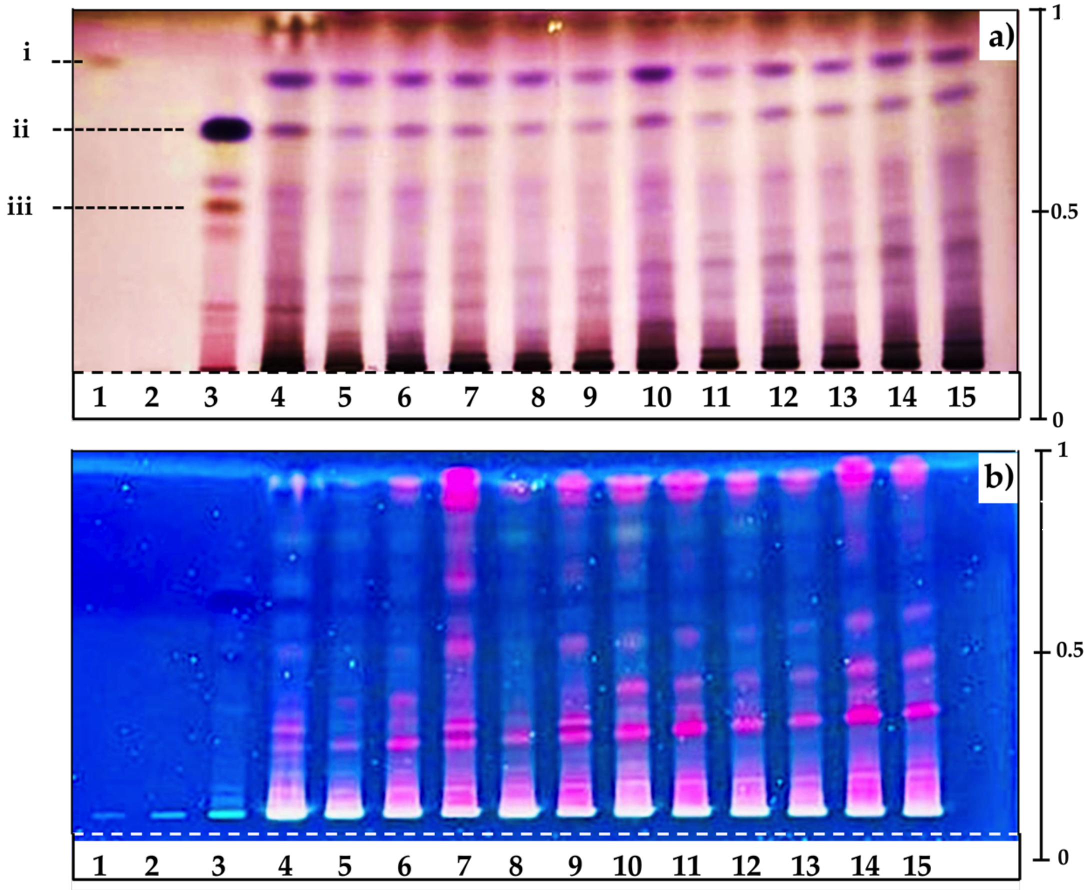

Figure Supplementary Data 9. HPTLC chromatograms of dichloromethane extracts of *B. conferta* after elicitation with H_2O_2. (**a**) at white light, (**b**) at UV 365 nm. Lanes 1-3 were standards: i) Bacchofertin, ii) oleanolic acid, and iii) kingidiol. Lanes: 4 (control), 5 (25 µM H_2O_2), and 6 (250 µM H_2O_2) at 0 h. Lanes: 7 (control), 8 (25 µM H_2O_2), and 9 (250 µM H_2O_2) at 9 h, lanes 10 (control), 11 (25 µM H_2O_2), and 12 (250 µM H_2O_2) at 24 h. Lanes: 13 (control), 14 (25 µM H_2O_2), and 15 (250 µM H_2O_2) at 48 h.
