# Supplementary figures and images for: Hydrogen Peroxide-Oxidative Signaling Enhances Biosynthesis of Specialized Metabolites in Baccharis conferta Kunth
Source: Int J Mol Sci. 2026 Mar 10;27(6):2544. doi: 10.3390/ijms27062544 (PMC13027281; doi:10.3390/ijms27062544)

# Supplementary Data S6. Alignment of DXS sequences reported in the NCBI database

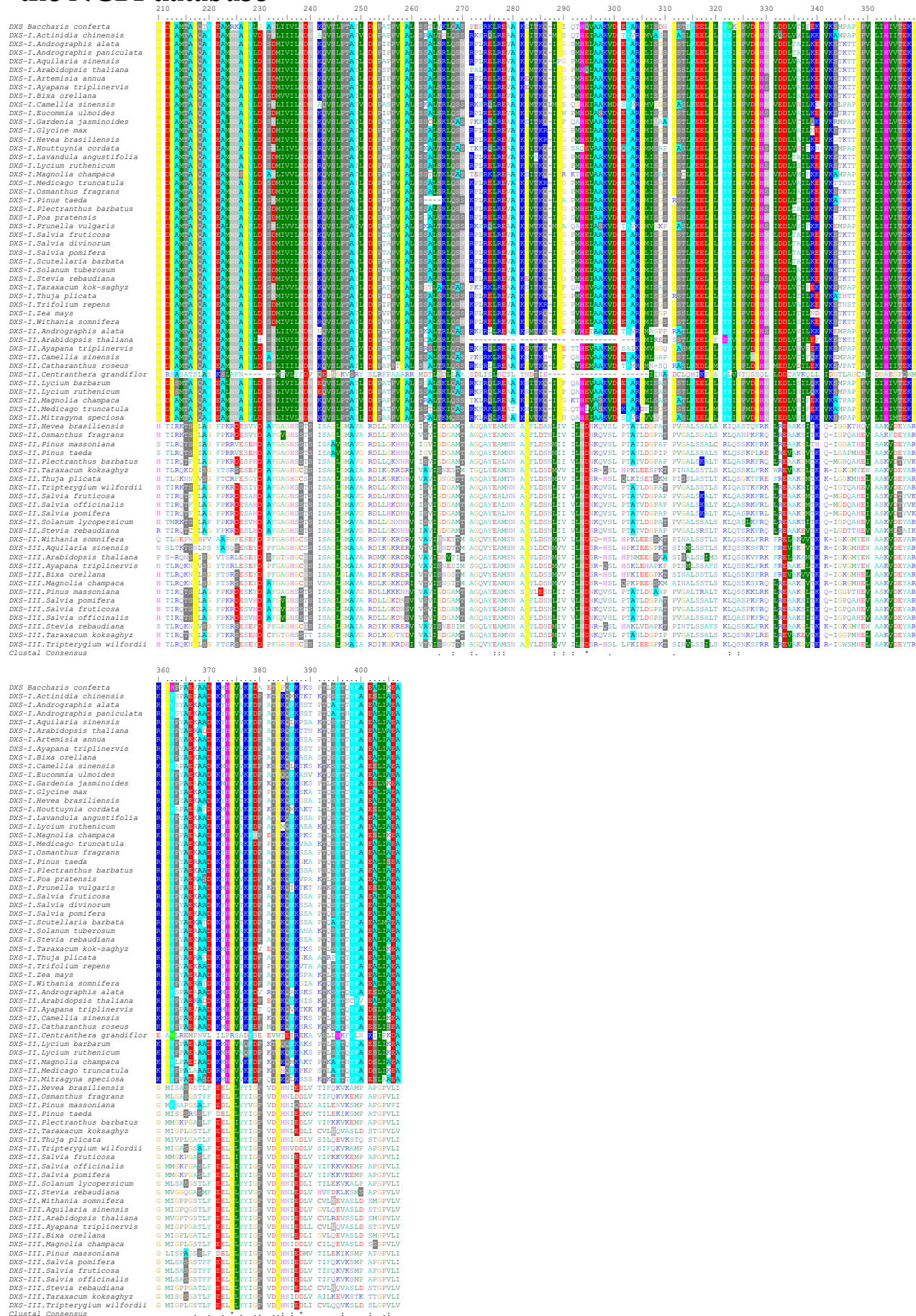

Supplement: Supplementary file 1 [file ijms-27-02544-s001.zip › Supplementary Data S6. Alignment of DXS sequences reported in the NCBI database.pdf]
